# CTHRC1^+^ cancer associated-fibroblasts promotes malignant progression of bladder cancer cells via inducing an immunosuppressive tumor microenvironment

**DOI:** 10.3389/fimmu.2026.1898002

**Published:** 2026-08-12

**Authors:** Miao Wang, Meng Lin, Yang Wang

**Affiliations:** 1 Laboratory Animal Center, West China School of Basic Medical Science & Forensic Medicine, Sichuan University, Chengdu, China; 2 School of Basic Medical Sciences, Zhengzhou University, Zhengzhou, China; 3 Sichuan Provincial People’s Hospital, Chengdu, China

**Keywords:** bladder cancer, cancer associated fibroblasts (CAFs), CTHRC1, macrophage polarization, Th2 cells

## Abstract

**Introduction:**

Bladder cancer remains a therapeutic challenge due to its high recurrence and metastasis rates. Cancer-associated fibroblasts (CAFs) are recognized as pivotal regulators of the tumor microenvironment (TME), however the specific contribution of CTHRC1^+^ CAFs subsets to bladder cancer progression remains poorly defined.

**Methods:**

CTHRC1^+^ CAFs were successfully isolated from human patient specimens and mouse xenograft models. To systematically evaluate the function of CTHRC1^+^ CAFs in bladder cancer, a comprehensive suite of assays was employed, including CCK-8, Transwell, murine xenograft models, ELISA, flow cytometry, and Western blotting.

**Results:**

We found that CTHRC1 was predominantly expressed in CAFs, and CTHRC1^+^ CAFs promoted cell growth, migration and invasion of bladder cancer cells both *in vitro* and *in vivo*. Moreover, CTHRC1^+^ CAFs fostered an immunosuppressive tumor microenvironment by driving M2 macrophage polarization and Th2 differentiation, which subsequently suppressed the antitumor activity of CD8^+^ T cells. Mechanistically, CTHRC1 activated the PI3K/Akt/NF-κB pathway in CAFs and induced the secretion of various immunomodulatory cytokines, which regulated M2 macrophage polarization and Th2 differentiation.

**Conclusions:**

These findings identify CTHRC1^+^ CAFs as key promoters of bladder cancer progression and immune evasion, suggesting CTHRC1 as a potential prognostic biomarker for bladder cancer.

## 1 Introduction

Bladder cancer represents a globally prevalent and therapeutically intractable urogenital malignancy (1). According to global cancer statistics, approximately 573,000 new cases were diagnosed in 2020, and this burden is projected to double by 2040 if current trends continue. Clinically, bladder cancer is classified into two principal subtypes: non-muscle-invasive bladder cancer (NMIBC) and muscle-invasive bladder cancer (MIBC) (2). Although NMIBC accounts for 70-80% of cases at diagnosis and exhibits a favorable 5-year survival rate (>85-90%), it is afflicted by a high recurrence rate exceeding 50% within 5 years (3). In stark contrast, MIBC constitutes 20-30% of cases and exhibits marked aggressiveness, high metastatic potential, and a dismal median survival of merely ~15 months following metastasis. Bladder cancer incidence varies markedly by geography, socioeconomic status, and sex, ranking sixth in males versus seventeenth in females (4). Etiologically, bladder cancer is strongly linked to environmental carcinogens like cigarette smoking and aromatic amines. The TME of bladder cancer represents a complex and dynamic ecosystem defined by the sophisticated crosstalk between malignant urothelial cells and a diverse repertoire of stromal components, including fibroblasts, endothelial cells, immune infiltrates, and the extracellular matrix (5). Consequently, a comprehensive understanding of the complexities governing the bladder tumor microenvironment is pivotal for elucidating the mechanisms underlying the high recurrence rates and the frequent therapeutic resistance observed in clinical practice.

Cancer-associated fibroblasts (CAFs) have emerged as pivotal components of the tumor microenvironment (TME), driving diverse pathological processes including tumor progression, metastatic dissemination, therapeutic resistance, and immune evasion (6, 7). Within the bladder tumor microenvironment, CAFs have emerged as dominant and functionally influential components of the tumor stroma. For example, PDGFRα^+^ ITGA11^+^ CAFs facilitate lymphatic metastasis of bladder cancer via recognizing ITGA11 surface receptor SELE on lymphatic endothelial cells (8). Single-cell RNA sequencing has identified MMP11^+^ CAFs as key drivers of angiogenesis and bladder cancer progression by regulating the migration of ESM1^+^ tip endothelial cell clusters (9). Given their profound involvement in fostering tumor advancement and circumventing immune surveillance, CAFs represent compelling targets for novel therapeutic strategies. For instance, NNMT, an NAD^+^ metabolism enzyme expressed in CAFs, promotes bladder cancer progression and induces resistance to anti-PD-1/PD-L1 immunotherapy by orchestrating the recruitment of tumor-associated macrophages. Therefore, a comprehensive elucidation of the molecular intricacies governing CAF biology is an absolute necessity for advancing future therapeutic strategies.

Collagen Triple Helix Repeat Containing 1 (CTHRC1) is an evolutionarily conserved secretory glycoprotein (10). Under physiological conditions, CTHRC1 expression is strictly confined to the bone matrix and periosteum in healthy adults, where it contributes to normal tissue repair and inhibits excessive fibrosis (11). However, under pathophysiological conditions such as tissue injury, fibrosis, or malignancy, CTHRC1 becomes markedly dysregulated and functions as an oncogenic driver (12). By activating key signaling cascades such as TGF-β, Wnt, FAK, and PI3K/AKT and extensively remodeling the tumor immune microenvironment, CTHRC1 promotes all stages of cancer progression, spanning from initial proliferation to distant metastasis (13). For example, single-cell and spatial transcriptome profiling of colorectal cancer demonstrates that CTHRC1^+^ fibroblasts facilitate epithelial-mesenchymal transition via activating WNT5A/MSLN signaling axis (14). In hepatocellular carcinoma, CTHRC1 drives metastasis by interacting with TGF-β receptors to modulate macrophage polarization toward an M2 phenotype (15). Given its widespread upregulation across diverse malignancies and strong association with poor clinical outcomes, CTHRC1 represents a promising universal prognostic biomarker and therapeutic target, with particular relevance to bladder cancer.

In the present study, we sought to elucidate the biological role of CTHRC1 within the bladder cancer microenvironment. Our data indicated that CTHRC1 was predominantly expressed in CAFs of bladder cancer microenvironment. CTHRC1^+^ CAFs enhanced bladder cancer cell growth, migration and invasion, and induced M2 macrophage polarization and Th2 differentiation in xenograft models, thereby suppressing anti-tumor T cell activity. Our findings reveal a novel role for CTHRC1 in bladder cancer progression.

## 2 Materials and methods

### 2.1 Cell lines and reagents

The human bladder cancer cell lines T24, SW780, and J82, together with the murine bladder carcinoma cell line MB49, were procured from the Cell Bank of the Chinese Academy of Sciences (Shanghai, China) and iCell Bioscience Inc. (Shanghai, China), respectively. T24, SW780, J82 and MB49 cells were cultured in Dulbecco’s Modified Eagle’s Medium (DMEM, Gibco) supplemented with 10% fetal bovine serum (FBS, Sigma-Aldrich) and 1% penicillin-streptomycin (Sigma-Aldrich). All cultures were incubated at 37 °C in a humidified incubator containing 5% CO_2_.

### 2.2 Isolation of primary CAFs cell lines

The collection and use of human samples was approved by the Ethics Committee of Zhengzhou University (Approval No. 2023-KY-0625-003). Written informed consent was obtained from all patients prior to tissue collection. All animal experiments were approved by the Animal Care and Use Committee of Zhengzhou University (Approval No. ZZUEAC20230179). Primary murine cancer-associated fibroblast (M-CAF) lines were established following the subcutaneous inoculation of MB49 cells into C57BL/6 mice. Once tumor volumes reached approximately 800–1000 mm³, the animals were euthanized to enable the aseptic excision of tumor masses for subsequent M-CAF isolation. Concurrently, primary human CAFs and paired normal fibroblasts (NFs) were derived from fresh bladder cancer resection specimens and adjacent non-tumor mucosal tissues, respectively. The isolation of both CAFs and NFs was performed according to established protocols (16, 17). Briefly, tissues were minced into ~1 mm³ fragments and enzymatically dissociated in 10 mL PBS containing 400 IU/mL collagenase I, 100 IU/mL hyaluronidase and 10% FBS at 37 °C for 1 hours under gentle agitation. Digestion was quenched with complete medium (DMEM/F12, 10% FBS) and then centrifuged at 300 × g for 5 minutes. Pellets were resuspended and plated in 10 cm culture dishes. After 2–4 hours, non-adherent cells were washed away with PBS to enrich adherent stromal cells. Thereafter, cultures were maintained at 37 °C in a humidified incubator containing 5% CO_2_, with the culture medium refreshed every 2–3 days. To maintain phenotypic stability, only CAFs within 10 passages were used for subsequent experiments. The identity of these cells was confirmed by the expression of established fibroblast markers.

### 2.3 Plasmid constructs

For gain-of-function studies, human or mouse CTHRC1 coding sequences were cloned into the pCDH vector (Addgene, USA) to achieve ectopic overexpression, with the empty pCDH vector serving as the negative control. Conversely, for loss-of-function assays, two distinct short hairpin RNAs (shCTHRC1–1 and shCTHRC1-2) targeting both human and mouse CTHRC1 were designed and inserted into the PLKO.1 vector. A non-targeting sequence was cloned into PLKO.1 as negative control (shCtrl). The shRNA sequences used were as follows: shCTHRC1-1: 5’-GCTGA ATGTT CAGGA CCTCTT-3’; shCTHRC1-2: 5’-GGCAT AGATC TTGGG AAAAT TGC-3’; shCtrl: 5’-TTCTC CGAAC GTGTC ACGTTT-3’.

### 2.4 Western blot

Total protein was extracted from tissue specimens or cultured cells using RIPA lysis buffer (Beyotime) supplemented with protease and phosphatase inhibitor cocktails (Roche). Subsequently, the homogenates were centrifuged at 12,000 rpm for 10 min at 4 °C and the supernatant protein concentration was quantified using a BCA assay kit (Pierce). Equal amounts of protein (30 μg) were separated on 10% SDS-PAGE and electro-transferred onto PVDF membranes (Millipore). Next, membranes were blocked with 5% non-fat milk for 1 h at room temperature and incubated with primary antibodies at 4 °C overnight. Following TBST washes, blots were probed with secondary antibodies for 1 h at room temperature. Finally, Immunoreactive bands were visualized using ECL substrates (Thermo Fisher) and imaged with a ChemiDoc XRS system (Bio-Rad). Densitometric analysis was performed using ImageJ software. The primary antibodies used were as follows: α-SMA (Proteintech #67735-1-Ig), FAP (Invitrogen #PA5-99313), Vimentin (Proteintech #60330-1-Ig), FSP-1 (Proteintech #16105-1-AP), PDGFRβ (Proteintech #13449-1-AP), GAPDH (Cell Signaling Technology #97166), CTHRC1 (Proteintech #16534-1-AP), p-PI3K (T458) (Invitrogen #MA5-36955), PI3K (Cell Signaling #4257), p-Akt (S473) (Cell Signaling Technology #9271), Akt (Cell Signaling Technology #9272), p-p65 (S536) (Cell Signaling Technology #3031), p65 (Cell Signaling Technology #8242).

### 2.5 Quantitative real-time polymerase chain reaction

Total RNA was extracted using TRIzol reagent (Takara, Japan), and its purity was assessed by determining A260/A280 ratios. Subsequently, 1 μg of total RNA was reverse-transcribed into cDNA using the HiScript III Reverse Transcriptase (Vazyme). Quantitative real-time PCR amplification was conducted on a QuantStudio™ 5 Real-Time PCR System (Applied Biosystems, USA) with the SYBR Green/ROX PCR master mix (Invitrogen, USA). Relative gene expression levels were quantified using the 2^−ΔΔCt^ method, with GAPDH serving as the endogenous reference gene. The specific primer sequences used in this study are provided in **Supplementary Table 1**.

### 2.6 Cell viability assay

CTHRC1-overexpressing or knockdown CAFs, along with their corresponding control cells, were seeded in the upper chambers of transwells (Falcon) at a density of 2 × 10^4^ cells per well. Concurrently, bladder cancer cells (5 × 10^4^ cells) were plated in the lower chambers of 24-well plates to establish a co-culture system. To assess cell proliferation, 10 μL of CCK-8 reagent (Beyotime, China) was added to each well at predetermined time points (0, 2, 4, and 6 days post-co-culture), followed by incubation at 37 °C for 2 h. The absorbance at 450 nm was subsequently measured using a microplate reader, and cell proliferation curves were generated based on OD values.

### 2.7 Colony formation assay

CTHRC1-overexpressing or knockdown CAFs alongside their respective control cells (2 × 10^4^ cells) were seeded in the upper chambers of transwells, while T24, SW780, J82, and MB49 bladder cancer cells (2 × 10³ cells/well) were plated in the lower compartments. Following a co-culture period of 7–10 days, the colonies were fixed with 4% paraformaldehyde for 15–20 minutes and stained with 0.1% crystal violet. The number of colonies was quantified to determine the colony formation rate for each cell line.

### 2.8 Transwell migration and Matrigel invasion assay

The effect of CTHRC1-modulated CAFs on the migratory and invasive capabilities of bladder cancer cells was evaluated using Transwell chambers. Briefly, T24, SW780, J82, and MB49 cells (3 × 10^4^ cells/well) suspended in serum-free DMEM were seeded into the upper chambers, while CTHRC1-overexpressing or knockdown CAFs (5 × 10^4^ cells) were plated in the lower chambers containing medium supplemented with 10% FBS. For invasion assays, the upper surfaces of the membranes were pre-coated with Matrigel. Following incubation for 24 h (migration) or 36 h (invasion), non-migratory cells remaining on the upper membrane surface were removed, and cells that had traversed the membrane were fixed with 4% paraformaldehyde, stained with 0.1% crystal violet, and subsequently visualized under a microscope. All experiments were independently repeated three times. Triplicate independent experiments were performed.

### 2.9 Animal models

All animal experiments were approved by the Animal Care and Use Committee of Zhengzhou University (Approval No. ZZUEAC20230179). Mice were housed under specific pathogen-free conditions with free access to food and water, maintained on a 12-h light/dark cycle. All procedures complied with the Guide for the Care and Use of Laboratory Animals, and every effort was made to minimize animal suffering. Female Balb/c nude mice and C57BL/6 mice were obtained from Charles River (China). For the establishment of subcutaneous xenograft models, CTHRC1-modulated CAFs were co-injected with T24, SW780, or MB49 tumor cells at a 1:1 ratio (1 × 10^6^ cells each) into the flanks of mice. Tumor growth was monitored every 2–3 days. Tumor length (L) and width (W) were measured using electronic calipers, and tumor volume was calculated as V = (L × W²)/2. At the experimental endpoint, mice were euthanized by cervical dislocation, and the subcutaneous tumors were surgically excised, weighed, and collected for subsequent analyses.

### 2.10 Flow cytometry

To obtain single-cell suspensions for flow cytometry analysis, tissue samples were mechanically minced and enzymatically dissociated using 0.8 mg/mL Collagenase IV and 0.1 mg/mL DNase I (Sigma-Aldrich) at 37 °C for 45 minutes under constant rotation. The resulting suspensions were filtered through 70-μm nylon meshes (BD Biosciences) and subjected to erythrocyte lysis using ammonium-chloride-potassium (ACK) lysis buffer. For *in vitro* cultured cells, cells were digested with 0.25% Trypsin and filtered through 70-μm nylon meshes (BD Biosciences). To block non-specific binding, cells were pre-incubated with anti-Fc receptor blocking antibody. Surface antigen staining was subsequently performed on viable cells using fluorophore-conjugated antibodies targeting specific lineages. Intracellular staining was administered according to the manufacturer’s protocol. Briefly, cells were fixed and permeabilized using the Fixation/Permeabilization Solution Kit (BD bioscience, USA), then incubated with fluorochrome-conjugated antibodies. The flow cytometry antibody used in our study were: CD45 APC, CD3 PE, CD4 FITC, CD8 V450, CD11b Alexa Fluor™ 700, Ly6C PerCPCY5.5, Tim3 PE-CY7, PD-1 PerCP-eFluor™ 710, CD80 FITC, CD206 PE-CY7, IFN-γ PerCPCY5.5, IL-4 PE-CY7, GATA3 PE, T-bet APC, TNF-α PE-CY7, Ki67 eFluor™ 570 and GranB PE-CY7. The gating strategy for flow cytometry was shown in **Supplementary Figure 1**.

### 2.11 *In vitro* macrophage isolation and differentiation

Primary bone marrow-derived macrophages (BMDMs) were obtained by flushing the bone marrow from mouse femurs and tibiae. After erythrocyte lysis, the cells were cultured for 7 days in DMEM complete medium supplemented with 20 ng/mL GM-CSF to induce differentiation into M0-type macrophages. Concurrently, the human monocytic THP-1 cell line was differentiated into adherent M0 macrophages by stimulation with 50 ng/mL PMA for 24 hours. To evaluate macrophage polarization, M0-type BMDMs or THP-1 macrophages were co-cultured with CTHRC1-modulated CAFs under either direct or indirect conditions. For direct co-culture, macrophages and modified CAFs were seeded simultaneously into 24-well plates at a 1:1 ratio. To recapitulate the tumor microenvironment, cells were maintained in basic medium containing 10% FBS without the addition of exogenous polarizing cytokines. In the Transwell indirect co-culture system, modified CAFs were plated in the upper inserts while macrophages were seeded in the lower chambers, ensuring that macrophage polarization was driven solely by paracrine factors from the CAFs. After co-culture for 48–72 hours, macrophages were collected to assess the expression levels of M1 and M2 phenotypic markers via flow cytometry or RT-PCR.

### 2.12 *In vitro* T cell isolation and differentiation

Naïve T cells (CD62L^hi^CD44^lo^) were isolated from human or mouse peripheral blood using Ficoll reagent (Amersham, USA) according to the manufacturer’s protocol. To examine the impact of CAFs on T cell differentiation, naïve T cells were activated with anti-CD3 (2 μg/mL), anti-CD28 (2 μg/mL), and IL-2 (20 ng/mL) for 5 days under two distinct conditions: direct physical contact with CAFs or indirect co-culture using a transwell system. To specifically assess the influence of CAFs on Th1 polarization, naïve T cells were co-cultured with CAFs under both direct contact and transwell-separated settings, in the presence of anti-IL-4 (10 μg/mL) and IL-12 (20 ng/mL) in addition to the aforementioned stimulatory antibodies and cytokine. Likewise, for Th2 polarization, co-culture was conducted under both direct contact and transwell-separated settings in the presence of anti-IFN-γ (10 μg/mL) and IL-4 (20 ng/mL) for five days.

### 2.13 Single-cell analysis

Single-cell RNA sequencing analysis was conducted utilizing R (version 4.2.3) in conjunction with the Seurat package (version 5.0.0). Two publicly available single-cell RNA sequencing datasets (18, 19) were first underwent preprocessing, during which Seurat objects were constructed via the CreateSeuratObject function, followed by the incorporation of mitochondrial metrics through the PercentageFeatureSet function. Stringent quality control thresholds were applied to retain high-quality cells, specifically requiring an nFeature_RNA count between 200 and 7,500, a mitochondrial gene percentage (percent. mt) below 5%, and a hemoglobin gene percentage (percent. HB) below 3%, while concurrently excluding erythrocyte-related and mitochondria-associated genes. These two datasets were subsequently integrated using the Merge function. Standard data processing workflows were executed, encompassing the identification of highly variable genes via the NormalizeData and FindVariableFeatures functions, data scaling with ScaleData, and dimensionality reduction through principal component analysis (PCA) and t-distributed stochastic neighbor embedding (t-SNE). Finally, cell subpopulations were annotated according to the marker genes in **Supplementary Table 2**.

### 2.14 ELISA assay

Concentrations of IFN-γ, IL-4, TNF-α, TGF-β, IL-6, IL-10, CCL-2, and CXCL2 within tumor tissues or cell culture supernatants were determined using commercial ELISA kits (Abcam) following the manufacturer’s recommended protocols. Briefly, freshly harvested tumors were weighed and homogenized in ice-cold lysis buffer utilizing a mechanical tissue grinder. After centrifugation at 12,000 × *g* for 15 min at 4 °C, the supernatants were collected. Total protein concentrations were subsequently determined using a BCA assay to normalize the cytokine levels. For the ELISA procedure, samples were dispensed into antibody-precoated microplates and incubated at ambient temperature for 1 h. Subsequent to five washes with PBS, biotinylated detection antibodies, streptavidin-HRP conjugates, substrate solution, and stop solution were added sequentially in strict accordance with the manufacturer’s protocol. Finally, the absorbance was measured at 450 nm using a microplate reader, with each sample analyzed in triplicate.

### 2.15 Statistical analysis

Statistical analyses were performed using GraphPad Prism version 9 (GraphPad Software, San Diego, CA, USA). Data are presented as the mean ± standard deviation (SD). Two-group comparisons were analyzed using the two-tailed Student’s t-test, while multigroup comparisons were assessed by one-way ANOVA with Bonferroni *post hoc* testing. A p-value less than 0.05 was considered indicative of a statistically significant difference across all tests.

## 3 Results

### 3.1 Identification of CTHRC1^+^ cancer-associated fibroblasts in bladder cancer

**Figure 1:**
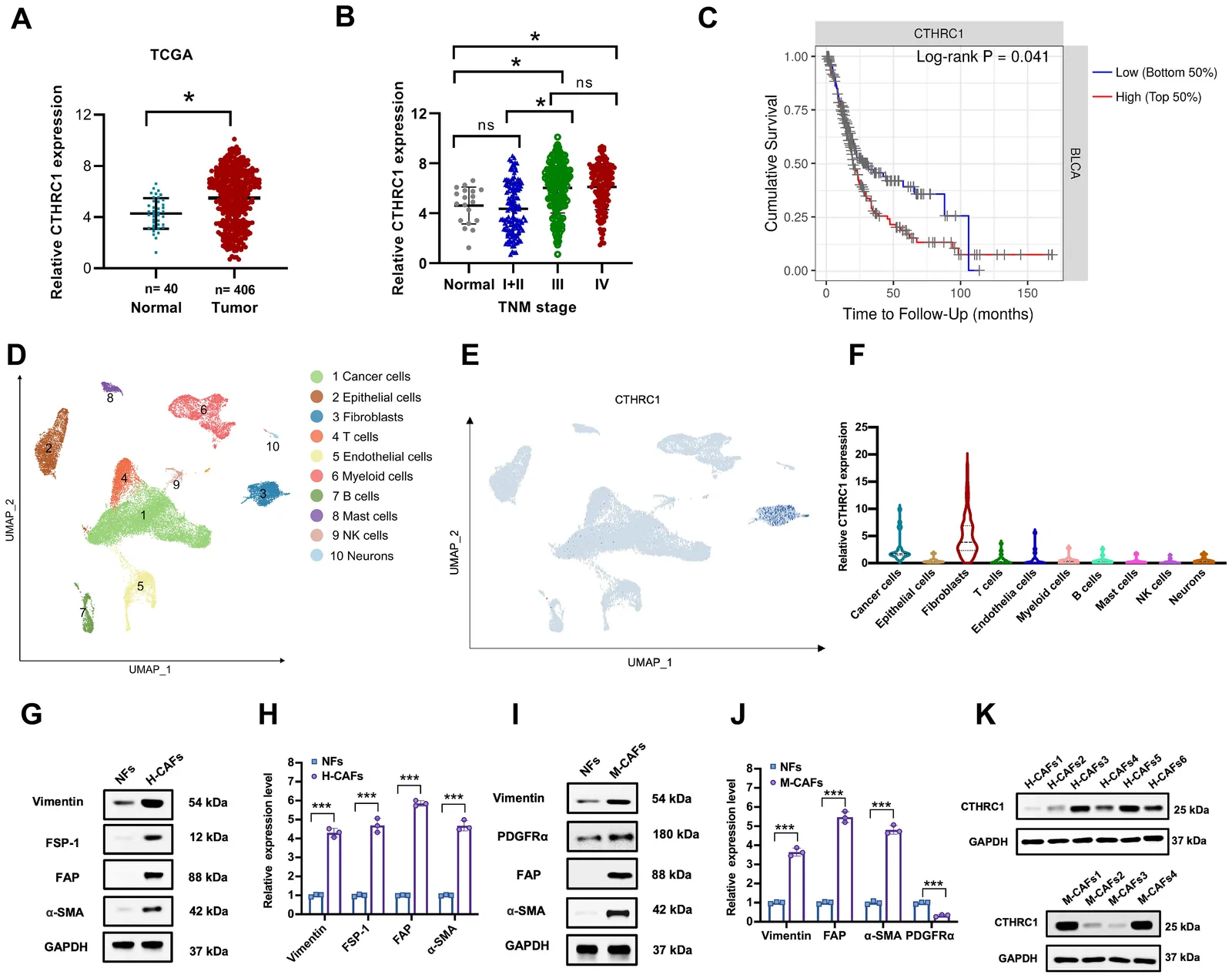
CTHRC1 is overexpressed in bladder cancer and primarily expressed in CAFs. **(A)** Analysis of the TCGA database revealed that CTHRC1 mRNA expression was significantly upregulated in bladder tumor tissues compared to normal tissues. **(B)** CTHRC1 mRNA expression levels were found to increase with advancing TNM stage in bladder cancer patients. **(C)** Kaplan-Meier survival analysis of TCGA data indicated that high CTHRC1 expression was associated with poor overall survival in bladder cancer patients (BLCA). **(D)** UMAP visualization of single-cell RNA sequencing data from bladder tumors, showing the distribution of 10 different cell types, including cancer cells (Cluster 1), epithelial cells (Cluster 2), fibroblasts (Cluster 3), T cells (Cluster 4), endothelial cells (Cluster 5), myeloid cells (Cluster 6), B cells (Cluster 7), mast cells (Cluster 8), NK cells (Cluster 9), and neurons (Cluster 10). **(E)** FeaturePlot showing that CTHRC1 expression is predominantly enriched in fibroblast clusters (Cluster 3). **(F)** Violin plot showing CTHRC1 expression across cell-types. **(G, H)**. Human bladder cancer CAFs (H-CAFs) exhibited significantly higher expression of CAF markers (Vimentin, FSP-1, FAP, and α-SMA) compared to normal fibroblasts (NFs), as confirmed by both Western blot (protein level) and qRT-PCR (mRNA level). **(I, J)**. Mouse bladder tumor CAFs (M-CAFs) showed significantly increased expression of Vimentin, FAP, α-SMA, and PDGFRα compared to normal fibroblasts (NFs), as confirmed by both Western blot (protein level) and qRT-PCR (mRNA level). **(K)** Western blot analysis of CTHRC1protein levels in various H-CAFs and M-CAFs strains, confirming heterogeneous CTHRC1expression within the CAF population. Data are presented as mean ± SD. *p < 0.05, ***p < 0.001 indicate statistical significance.

Substantial literature supports the prevalent upregulation of CTHRC1 within the CAF compartment of numerous cancers (20). Leveraging TCGA cohorts, we observed significant CTHRC1 overexpression across a broad spectrum of cancers (**Supplementary Figure 2**). Within the TCGA bladder cancer cohort, high CTHRC1 expression was strongly associated with advanced tumor progression and reduced patient survival rates (**Figures 1A–C**). Further analysis of publicly available single-cell RNA sequencing datasets (18, 19) revealed that CTHRC1 is primarily enriched in bladder cancer-derived CAFs (**Figures 1D–F**). To explore the potential role of CTHRC1 in CAFs, we established primary CAF cultures from human bladder cancer specimens (H-CAFs) and murine bladder tumor xenografts (M-CAFs). H-CAFs exhibited marked upregulation of Vimentin, FSP-1, FAP, and α-SMA compared to normal fibroblasts (NFs), verifying successful CAF isolation (**Figures 1G, H**). Consistent with this phenotype, M-CAFs displayed elevated expression of Vimentin, FAP, α-SMA, and PDGFRα, confirming the effective derivation of murine CAFs from tumor xenografts (**Figures 1I, J**). We established a panel of primary CAF cultures, comprising six human strains (H-CAFs1-6) and four murine strains (M-CAFs1-4). Subsequent Western blot analysis revealed heterogeneous CTHRC1 expression across these isolates: H-CAFs3, H-CAFs5, M-CAFs1, and M-CAFs4 exhibited high CTHRC1 levels, whereas H-CAFs1, H-CAFs2, M-CAFs2, and M-CAFs3 displayed minimal expression (**Figure 1K**). Collectively, these findings confirm the presence of CTHRC1^+^ cancer-associated fibroblasts in bladder cancer.

### 3.2 CTHRC1^+^ CAFs promote the proliferation, migration, and invasion of bladder cancer cells *in vitro* and *in vivo*

**Figure 2:**
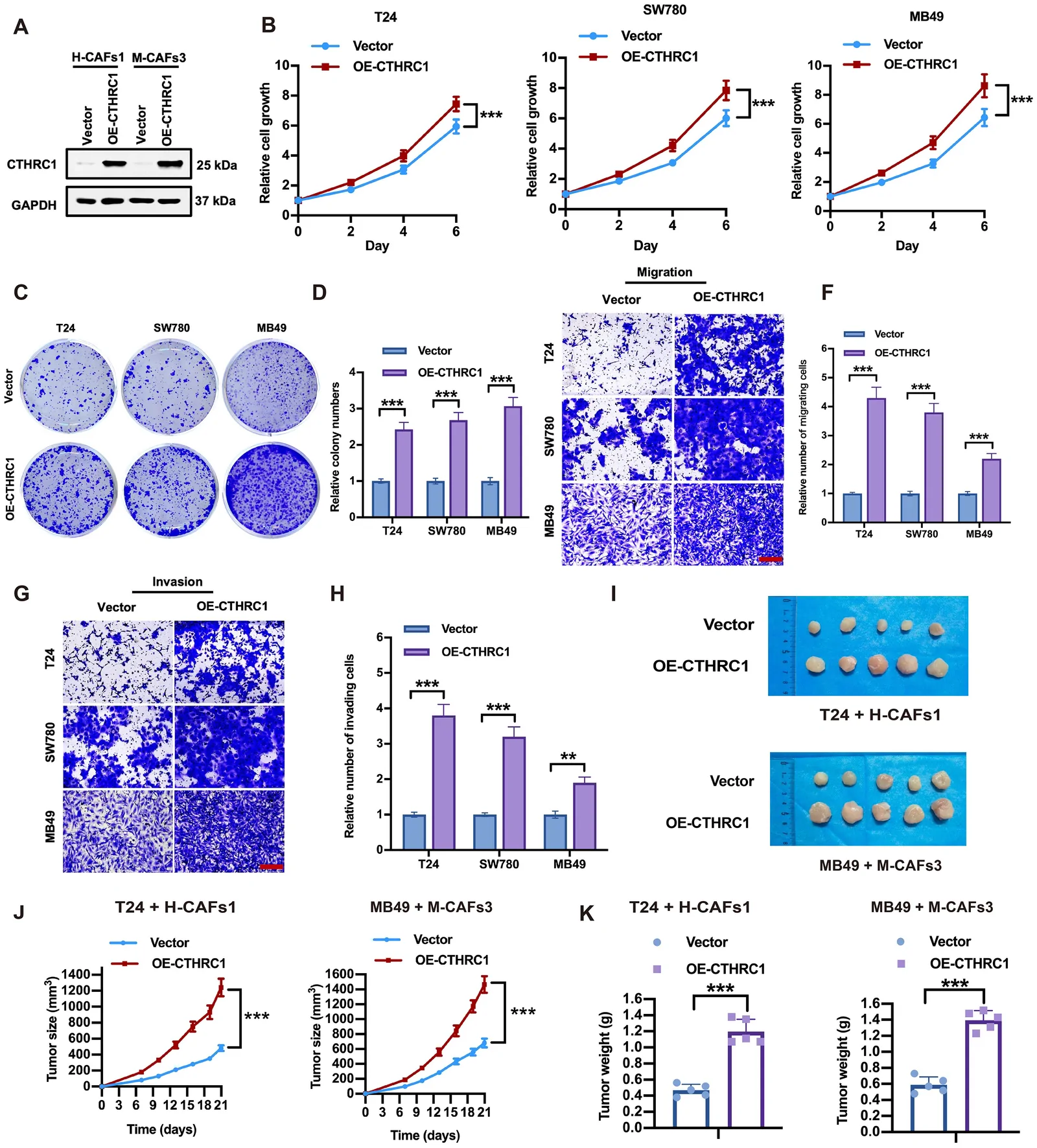
Overexpression of CTHRC1 promote the growth and metastasis of bladder cancer cells both in vitro and in vivo. **(A)** Western blot analysis confirmed the successful overexpression of CTHRC1 in H-CAFs1 and M-CAFs3 (OE-CTHRC1) compared to vector control groups. **(B)** CCK-8 cell viability assays demonstrated that co-culture with OE-CTHRC1 CAFs in a Transwell system significantly enhanced the proliferation of human T24 and SW780 and mouse MB49 bladder cancer cells compared to the Vector group. **(C, D)**. Colony formation assays showed that co-culture with OE-CTHRC1 CAFs in a Transwell system significantly increased the colony-forming ability of bladder cancer cells. **(E-H)**. Transwell migration and invasion assays revealed that co-culture with OE-CTHRC1 CAFs significantly promoted the migratory and invasive capacity of T24, SW780, and MB49 cells. Scale bar, 500 μm. **(I-K)**. T24 and H-CAFs1 cells (1: 1) were mixed together and subcutaneously implanted into Balb/c nude mice, while MB49 and M-CAFs3 cells (1: 1) were mixed together and subcutaneously implanted into C57BL/6 mice. Tumor were allowed to grow for three weeks. Representative images of tumor xenografts **(I)**, tumor growth curves **(J)** and tumor weight **(K)** were shown. Data are presented as mean ± SD; **p < 0.01, ***p < 0.001 indicate statistical significance.

To evaluate the potential influence of CTHRC1^+^ CAFs on bladder cancer progression, we ectopically overexpressed CTHRC1 in CAFs and performed indirect Transwell co-culture with bladder cancer cells. Given their low endogenous CTHRC1 levels, H-CAFs1 and M-CAFs3 were selected for gain-of-function analyses. Successful ectopic overexpression of CTHRC1 in these cells was confirmed by Western blotting (**Figure 2A**). Subsequently, CTHRC1-overexpressing H-CAFs1 were co-cultured with human bladder cancer cell lines T24 and SW780, while CTHRC1-overexpressing M-CAFs3 were co-cultured with the murine bladder cancer cell line MB49. Compared with control cells transduced with empty vector (Vector), co-culture with CTHRC1-overexpressing CAFs (OE-CTHRC1) markedly enhanced the viability and clonogenic capacity of bladder cancer cells, as evidenced by cell viability and colony formation assays (**Figures 2B–D**). Furthermore, Transwell migration and invasion assays demonstrated that OE-CTHRC1 CAFs significantly potentiated the migratory and invasive properties of bladder cancer cells (**Figures 2E–H**). The tumor-promoting effect of CTHRC1^+^ CAFs was further validated *in vivo*. Specifically, mixtures of T24 cells with OE-CTHRC1 H-CAFs1 were subcutaneously implanted into 6-week-old Balb/c nude mice, whereas MB49 cells mixed with OE-CTHRC1 M-CAFs3 were inoculated into immunocompetent C57BL/6 mice. Compared with vector-transduced controls, CTHRC1-overexpressing CAFs significantly accelerated the *in vivo* tumorigenicity of T24 and MB49 cells (**Figures 2I–K**).

**Figure 3:**
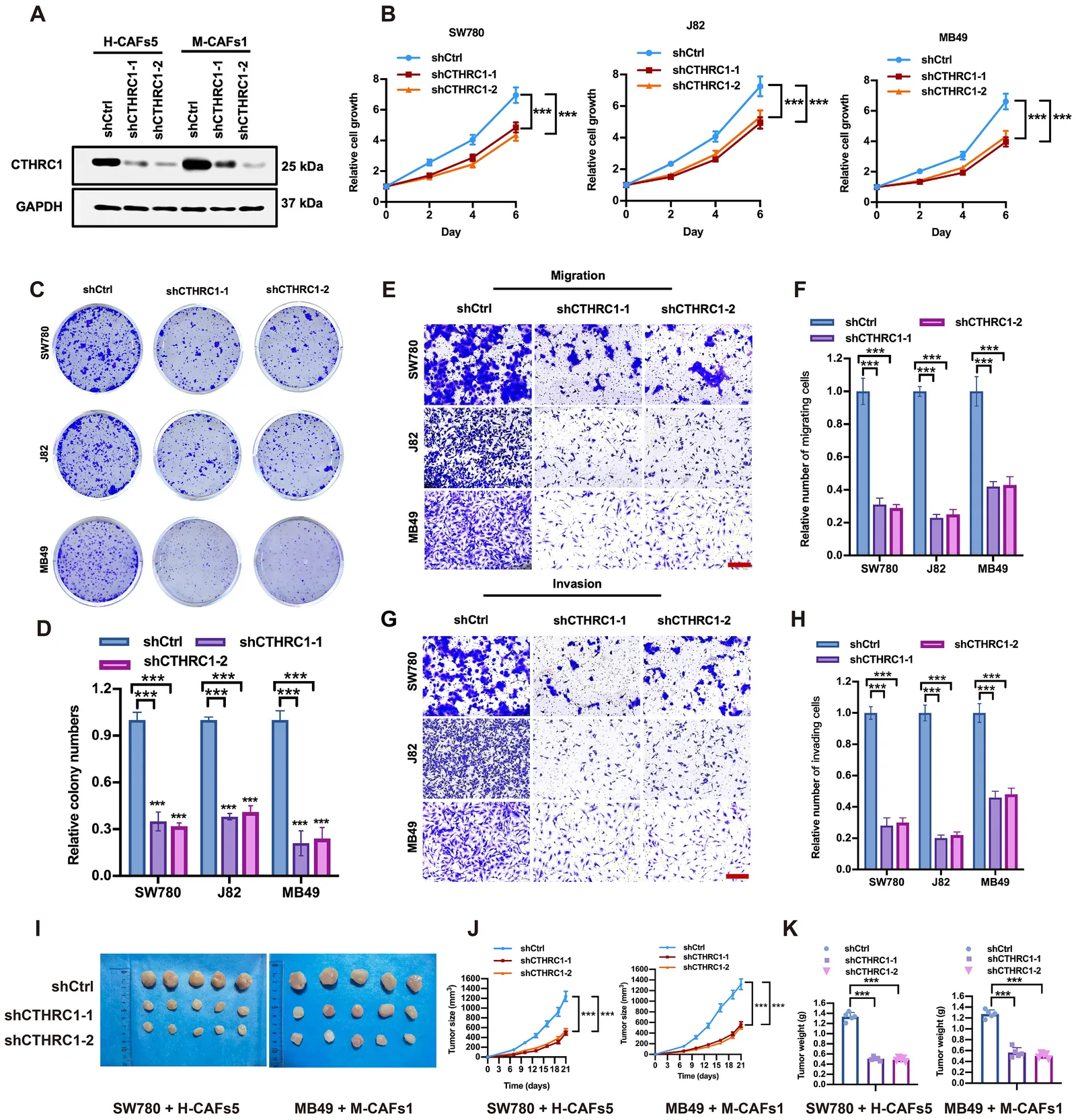
Knockdown of CTHRC1 inhibits the growth and metastasis of bladder cancer cells both *in vitro* and *in vivo*. **(A)** Western blot analysis confirmed the efficient knockdown of CTHRC1 in H-CAFs5 and M-CAFs1 (shCTHRC1-1 and shCTHRC1-2) compared to shCtrl groups. **(B)** CCK-8 cell viability assays demonstrated that co-culture with CTHRC1 knockdown CAFs in a Transwell system significantly inhibited the proliferation of human SW780 and J82 and mouse MB49 bladder cancer cells compared to the shCtrl group. **(C, D)**. Colony formation assays showed that co-culture with CTHRC1 knockdown CAFs in a Transwell system significantly decreased the colony-forming ability of bladder cancer cells. **(E-H)**. Transwell migration and invasion assays revealed that co-culture with CTHRC1 knockdown CAFs significantly suppressed the migratory and invasive capacity of SW780, J82, and MB49 cells. **(I-K)**, SW780 and CTHRC1 knockdown H-CAFs5 cells (1: 1) were mixed together and subcutaneously implanted into Balb/c nude mice, while MB49 and CTHRC1 knockdown M-CAFs1 cells (1: 1) were mixed together and subcutaneously implanted into C57BL/6 mice. Tumor were allowed to grow for three weeks. Representative images of tumor xenografts **(I)**, tumor growth curves **(J)** and tumor weight **(K)** were shown. Data are presented as mean ± SD; ***p < 0.001 indicate statistical significance.

To complement these gain-of-function findings, we next employed loss-of-function strategies to elucidate the biological impact of CTHRC1. Given their high endogenous CTHRC1 expression, H-CAFs5 and M-CAFs1 were selected for subsequent knockdown experiments. Two short hairpin RNAs (shCTHRC1–1 and shCTHRC1-2) targeting both human and mouse CTHRC1 were engineered and transduced into these cells, achieving efficient CTHRC1 depletion as confirmed by Western blotting (**Figure 3A**). In our study, CTHRC1-depleted H-CAFs5 cells were co-cultured with human bladder cancer cell lines SW780 and J82, while CTHRC1-depleted M-CAFs1 cells were co-cultured with mouse bladder cancer cell line MB49 using a transwell system. Bladder cancer cells co-cultured with CTHRC1-depleted CAFs (shCTHRC1–1 and shCTHRC1-2) exhibited substantially decreased viability and fewer colonies compared to those co-cultured with shCtrl CAFs (**Figures 3B–D**). In parallel, the migratory and invasive capacities of bladder cancer cells were notably reduced upon co-culture with CTHRC1-deficient CAFs in Transwell assays (**Figures 3E–H**). The *in vivo* relevance of CTHRC1 in CAFs was subsequently evaluated using subcutaneous xenograft models. Mixtures of SW780 cells with CTHRC1-depleted H-CAFs5 were implanted into 6-week-old Balb/c nude mice, while MB49 cells combined with CTHRC1-depleted M-CAFs1 were inoculated into immunocompetent C57BL/6 mice. Compared with shCtrl-transduced controls, CTHRC1 deficiency in CAFs significantly impaired their capacity to promote tumorigenesis (**Figures 3I–K**). Collectively, these findings demonstrate that CTHRC1^+^ CAFs drive the proliferation, migration, and invasion of bladder cancer cells across both *in vitro* and *in vivo* settings.

### 3.3 CTHRC1^+^ CAFs induce the infiltration of immunosuppressive M2 macrophage and Th2 cells in bladder cancer xenografts

**Figure 4:**
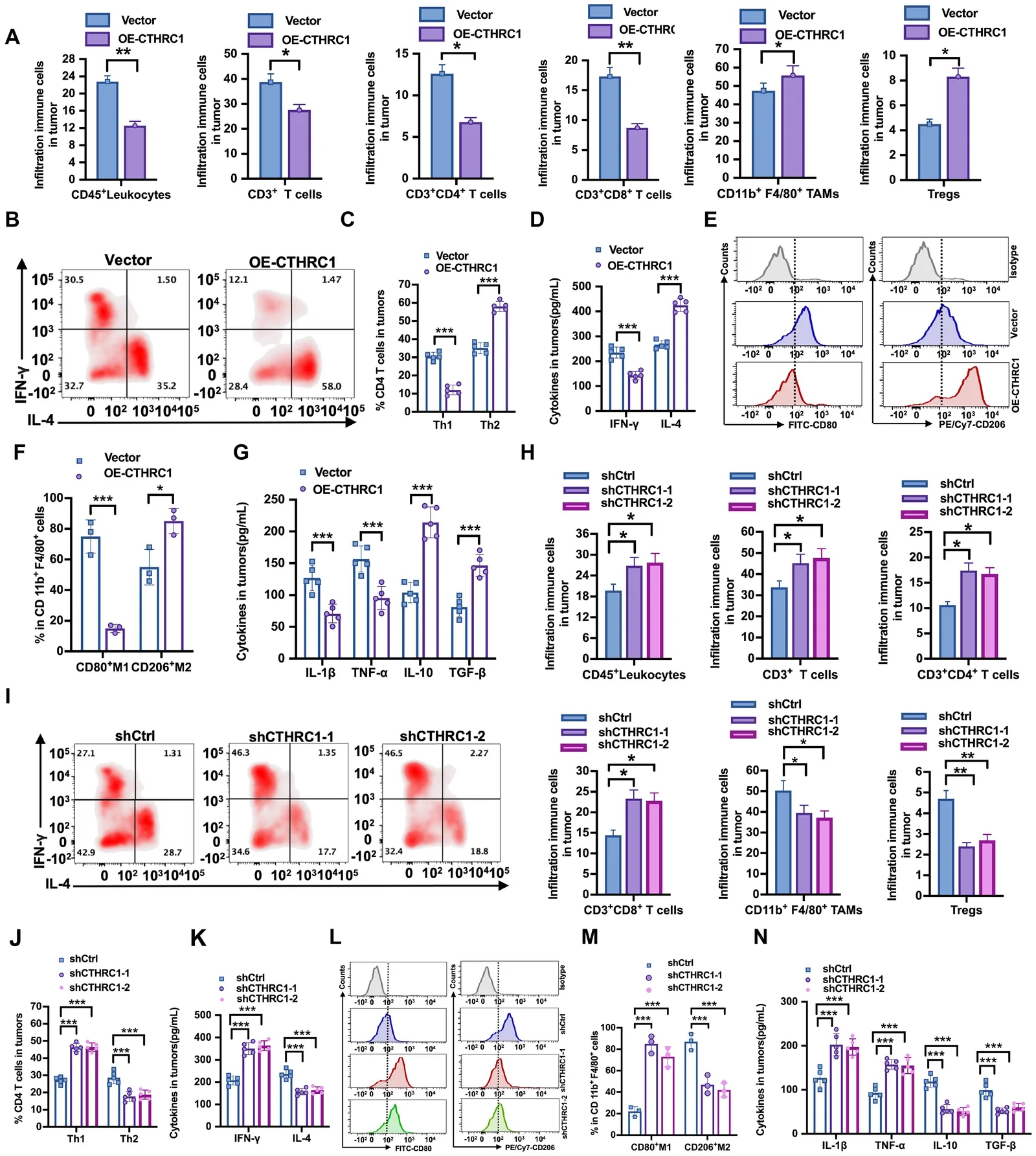
CTHRC1^+^ CAFs induce an immunosuppressive tumor microenvironment *in vivo*. **(A)**. Flow cytometric analysis of immune cell subsets in MB49 xenografts. CTHRC1-overexpression (OE-CTHRC1) CAFs reduced the infiltration of CD45^+^ leukocytes, CD3^+^ T cells, CD4^+^ T helper (Th) cells and CD8^+^ cytotoxic T (cyto T) cells, but promoted the infiltration of CD11b^+^ F4/80^+^ TAMs and Tregs. **(B, C)**. Flow cytometric analysis of Th1 (IFN-γ^+^) and Th2 (IL-4^+^) subsets in CD4^+^ Th cells of MB49 xenografts. **(D)**. ELISA assay indicated that CTHRC1-overexpression CAFs elevated IL-4 production while suppressed IFN-γ production in MB49 tumors. **(E, F)**. Flow cytometric analysis indicated that CTHRC1-overexpression CAFs increased CD206^+^ M2-like TAMs infiltration and decreased CD80^+^ M1-like TAMs infiltration in MB49 tumors. **(G)**. ELISA assay indicated that CTHRC1-overexpression CAFs suppressed pro-inflammatory (IL-1β and TNF-α) and enhanced anti-inflammatory (IL-10, TGF-β) cytokines production in MB49 tumors. **(H)**. Flow cytometric analysis of immune cell subsets in MB49 xenografts. CTHRC1 knockdown CAFs promoted the infiltration of CD45^+^, CD3^+^ T cells, CD4^+^ Th cells and CD8^+^ cyto T cells, but restrained the infiltration of CD11b^+^ F4/80^+^ TAMs and Tregs. **(I, J)**. Flow cytometric analysis of Th1 (IFN-γ^+^) and Th2 (IL-4^+^) subsets in CD4^+^ Th cells of MB49 xenografts, which indicated that CTHRC1 knockdown CAFs significantly promoted Th1 (IFN-γ^+^) differentiation while inhibiting Th2 (IL-4^+^) differentiation in CD4^+^ Th cells. **(K)**. ELISA assay indicated that CTHRC1 knockdown CAFs suppressed IL-4 while promoted IFN-γ production in MB49 tumors. **(L, M)**. Flow cytometry analysis indicated that CTHRC1 knockdown CAFs decreased CD206^+^ M2-like TAMs infiltration and increased CD80^+^ M1-like TAMs infiltration in MB49 tumors. **(N)**. ELISA assay indicated that CTHRC1 knockdown CAFs elevated pro-inflammatory (IL-1β, IL-6, TNF-α) and reduced anti-inflammatory (IL-10, TGF-β) cytokines production in MB49 tumors. Data are presented as mean ± SD; *p < 0.05, **p < 0.01, ***p < 0.001 indicate statistical significance.

As a secretory glycoprotein, accumulating evidence has established that CTHRC1 plays a pivotal role in remodeling the tumor microenvironment across various malignancies (15, 21, 22). Nevertheless, its specific impact on the bladder cancer microenvironment remains largely undefined. In this study, we leveraged the TIMER algorithm to assess the correlation between CTHRC1 expression and immune infiltrate abundance using TCGA bladder cancer cohort data. Bioinformatics analysis revealed a significant positive correlation between CTHRC1 levels and the infiltration of CD4^+^ T helper 2 (Th2) cells, macrophages, and regulatory T (Treg) cells (**Supplementary Figure 3**). To validate these findings *in vivo*, CTHRC1-overexpressing M-CAFs3 cells were admixed with MB49 cells and subcutaneously implanted into 6-week-old immunocompetent C57BL/6 mice. Subsequent flow cytometric analysis revealed that CTHRC1 overexpression suppressed the infiltration of CD45^+^ leukocytes, CD3^+^ T cells, CD4^+^ Th cells, and CD8^+^ CTLs, while enhancing the recruitment of CD11b^+^F4/80^+^ TAMs and Tregs (**Figure 4A**). Th1 and Th2 represent two principal subsets of CD4^+^ T cells, classically defined by their distinct effector cytokine profiles. Th1 cells are characterized by the production of IFN-γ, whereas Th2 cells are defined by IL-4 secretion. Consistent with these immunophenotypic signatures, we delineated Th1 cells as CD3^+^CD4^+^IFN-γ^+^IL-4^−^ and Th2 cells as CD3^+^CD4^+^IFN-γ^−^IL-4^+^ for subsequent analysis. Strikingly, tumors coexisting with CTHRC1-overexpressing M-CAFs3 exhibited a pronounced shift towards Th2 differentiation coupled with a concomitant suppression of Th1 differentiation (**Figures 4B, C**). This phenotypic skew was corroborated by cytokine profiling, which showed elevated IL-4 and diminished IFN-γ production within these tumors (**Figure 4D**). We next investigated the impact of CTHRC1 on macrophage polarization, utilizing CD80 and CD206 as canonical markers for M1 and M2 macrophages, respectively. CTHRC1-overexpressing M-CAFs3 facilitated the polarization of TAMs towards an immunosuppressive CD206^+^ M2 phenotype while concurrently restraining the CD80^+^ M1 phenotype (**Figures 4E, F**). Consistent with this M2-skewed polarization, CTHRC1-overexpressing M-CAFs3 attenuated the production of pro-inflammatory cytokines (IL-1β and TNF-α) while augmenting the secretion of anti-inflammatory cytokines (IL-10 and TGF-β) within MB49 tumors (**Figure 4G**). To substantiate these observations, we performed loss-of-function experiments using CTHRC1-depleted M-CAFs1 cells. In contrast to shCtrl-transduced M-CAFs1, CTHRC1 knockdown in M-CAFs1 enhanced CD45^+^ leukocyte, CD3^+^ T cell, CD4^+^ Th cell, and CD8^+^ CTL infiltration, while suppressing CD11b^+^F4/80^+^ TAM and Treg accumulation in MB49 tumors (**Figure 4H**). Meanwhile, CTHRC1 depletion in M-CAFs1 shifted the T cell balance toward a Th1-polarized phenotype, markedly facilitating Th1 differentiation and IFN-γ production, while concomitantly attenuating Th2 differentiation and IL-4 synthesis in MB49 tumors (**Figures 4I–K**). Furthermore, CTHRC1 depletion in M-CAFs1 curtailed the infiltration of CD206^+^ M2 TAMs and the production of anti-inflammatory cytokines (IL-10 and TGF-β), while potentiating the recruitment of CD80^+^ M1 TAMs and the secretion of pro-inflammatory cytokines (IL-1β, IL-6, TNF-α) within MB49 tumors (**Figures 4L–N**). Collectively, these data demonstrate that CTHRC1^+^ CAFs orchestrate an immunosuppressive microenvironment in bladder cancer xenografts, characterized by the robust recruitment of M2 macrophages and Th2 cells.

### 3.4 CTHRC1^+^ CAFs facilitate Th2 cell differentiation and M2 macrophage polarization *in vitro*

**Figure 5:**
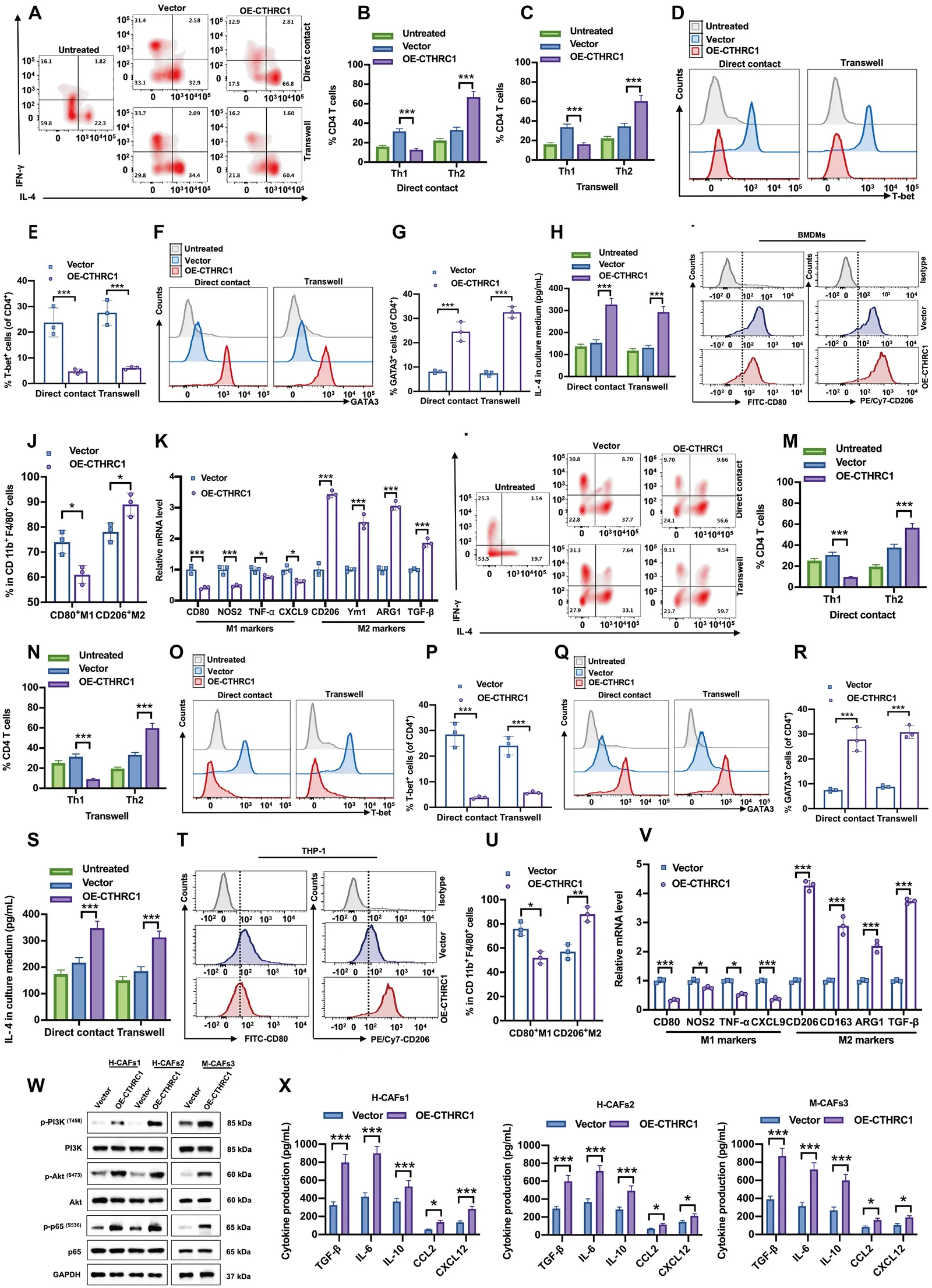
CTHRC1^+^ CAFs promote Th2 and M2 polarization via PI3K/Akt/NF-κB signaling in vitro. **(A-H)**. moues naïve T cells were activated and co-cultured with CTHRC1-overexpression M-CAFs3 cells indirectly using a transwell system or directly in 24-well plates. Flow cytometric analysis indicated that CTHRC1-overexpression CAFs suppressed Th1 (IFN-γ^+^) differentiation and promoted Th2 (IL-4^+^) differentiation of mouse naïve T cells **(A-C)**. CTHRC1-overexpression CAFs also increased the percentage of GATA3 (Th2 transcription factor) positive T cells and decreased the percentage of T-bet (Th1 transcription factor) positive T cells **(D-G)**. ELISA assay indicated that CTHRC1-overexpression CAFs increased IL-4 production in culture medium **(H)**. **(I-K)** CTHRC1-overexpression CAFs increased the percentage of CD206^+^ M2 macrophage and expression of M2 macrophage markers (CD206, Ym1, ARG1, TGF-β), and decreased the percentage of CD80^+^ M1 macrophage and expression M1 macrophage markers (CD80, NOS2, TNF-α, CXCL9) of mouse BMDMs. L-S. human naïve T cells were activated and co-cultured with CTHRC1-overexpression H-CAFs1 cells indirectly using a transwell system or directly in 24-well plates. Flow cytometric analysis indicated that CTHRC1-overexpression CAFs suppressed Th1 (IFN-γ^+^) differentiation and promoted Th2 (IL-4^+^) differentiation of human naïve T cells **(L-N)**. CTHRC1-overexpression CAFs also increased the percentage of GATA3 (Th2 transcription factor) positive T cells and decreased the percentage of T-bet (Th1 transcription factor) positive T cells **(O-R)**. ELISA assay indicated that CTHRC1-overexpression CAFs increased IL-4 production in culture medium **(S)**. **(T-V)** CTHRC1-overexpression CAFs increased the percentage of CD206^+^ M2 macrophage and expression of M2 macrophage markers (CD206, Ym1, ARG1, TGF-β), and decreased the percentage of CD80^+^ M1 macrophage and expression M1 macrophage markers (CD80, NOS2, TNF-α, CXCL9) of human THP1 cells. **(W)** Western blot analysis of the PI3K/Akt and NF-κB signaling pathways in human (H-CAFs1 and H-CAFs2) and mouse (M-CAFs3) CAFs following CTHRC1 overexpression. GAPDH served as the loading control. **(X)** Quantification of immunosuppressive cytokine secretion (TGF-β, IL-6, IL-10, CCL2, CXCL12) in the supernatants of H-CAFs1, H-CAFs2, and M-CAFs3 cells measured by ELISA. Data are presented as mean ± SD; *p < 0.05, **p < 0.01, ***p < 0.001 indicate statistical significance.

The immunomodulatory effects of CTHRC1^+^ CAFs on Th2 cell differentiation and M2 macrophage polarization were assessed *in vitro*. Naïve T cells were isolated from peripheral blood of C57BL/6 mice, then differentiated into Th1 and Th2 cells as previously described (23, 24). Activated naïve T cells were co-cultured with CTHRC1-overexpressing M-CAFs3 cells either indirectly via a transwell system or directly in 24-well plates. Compared with vector-transduced controls, CTHRC1-overexpressing M-CAFs3 cells significantly promoted Th2 differentiation while suppressing Th1 differentiation (**Figures 5A–C**). Given that T-bet and GATA-3 serve as lineage-defining transcription factors for Th1 and Th2 cells, respectively, we further examined their expression profiles. CTHRC1 overexpression elevated the frequency of GATA-3^+^ T cells and increased IL-4 secretion into the culture supernatant, whereas the proportion of T-bet^+^ T cells was markedly reduced (**Figures 5D–H**). Subsequently, bone marrow-derived macrophages (BMDMs) were isolated and polarized toward M1 or M2 phenotypes as previously described (25, 26). Co-culture with CTHRC1-overexpressing M-CAFs3 cells promoted M2 polarization, as evidenced by upregulated expression of M2-associated markers (CD206, Ym1, ARG1, and TGF-β), while concomitantly suppressing M1 polarization accompanied by downregulation of M1 markers (CD80, NOS2, TNF-α, and CXCL9) (**Figures 5I–K**). These findings were recapitulated in human systems. CTHRC1-overexpressing H-CAFs1 cells promoted Th2 differentiation in naïve human T cells (**Figures 5L–S**) and drove M2 polarization in THP-1 monocytes (**Figures 5T–V**). In addition, CTHRC1 overexpression markedly enhanced the phosphorylation of PI3K, Akt, and p65 in CAFs, thereby activating the PI3K/Akt/NF-κB signaling cascade (**Figure 5W**). Concurrently, CTHRC1 upregulation facilitated the secretion of a diverse array of cytokines, including TGF-β, IL-6, IL-10, CCL2, and CXCL12 (**Figure 5X**). To further validate the potential role of PI3K/Akt/NF-κB signaling cascade in CTHRC1-mediated effects, we used PI3K inhibitor (LY294002) and Akt inhibitor (MK-2206). Activated naïve T cells were co-cultured with CTHRC1-overexpressing M-CAFs3 cells via a transwell system, then treated with LY294002 or MK-2206. CTHRC1-overexpressing M-CAFs3 cells significantly increased the percentage of GATA3^+^ Th2 cells and IL-4 production, while reduced the percentage of T-bet^+^ Th1 cells, and this was abrogated by LY294002 or MK-2206 treatment (**Supplementary Figures 4A–E**). Moreover, CTHRC1 overexpression promoted M2 macrophage polarization and restrained M1 macrophage polarization, and this was abolished by LY294002 or MK-2206 treatment (**Supplementary Figures 4F–H**). Western blot analysis indicated that CTHRC1 overexpression activated PI3K/Akt/NF-κB signaling, but this was also reversed by LY294002 or MK-2206 treatment (**Supplementary Figure 4I**). CTHRC1 overexpression increased the production of TGF-β, IL-6, IL-10, CCL2, and CXCL12, but this was reversed by LY294002 or MK-2206 treatment (**Supplementary Figure 4J**). The above results indicated that PI3K/Akt/NF-κB signaling cascade acted a vital role in CTHRC1-mediated Th2 differentiation and M2 macrophage polarization.

**Figure 6:**
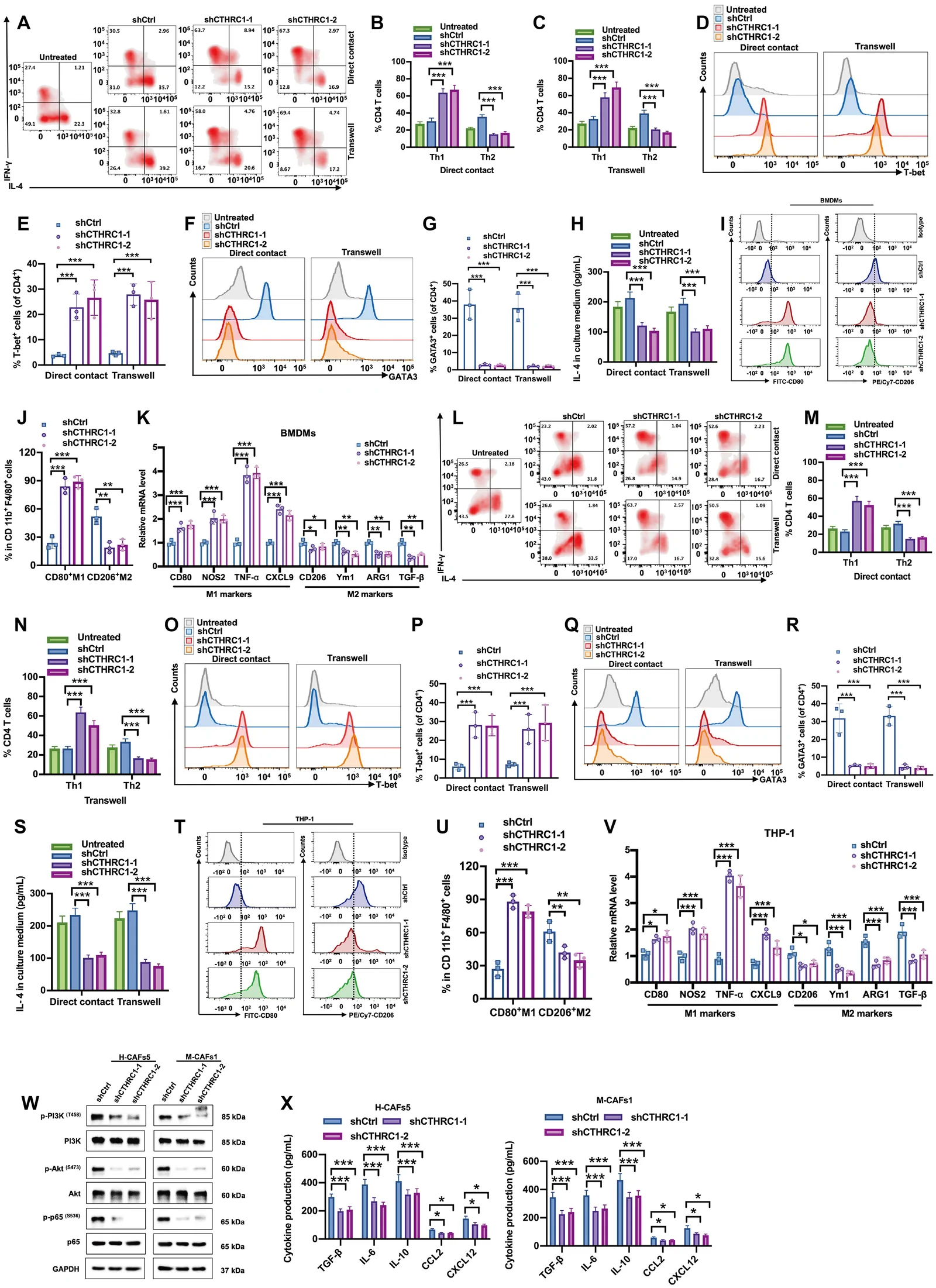
CTHRC1 knockdown in CAFs promote Th1 and M1 polarization via inhibiting the PI3K/Akt/NF-κB signaling pathway in vitro. **(A-H)**, moues naïve T cells were activated and co-cultured with CTHRC1 knockdown M-CAFs1 cells indirectly using a transwell system or directly in 24-well plates. Flow cytometric analysis indicated that CTHRC1 knockdown CAFs promoted Th1 (IFN-γ^+^) differentiation and suppressed Th2 (IL-4^+^) differentiation of mouse naïve T cells **(A-C)**. CTHRC1 knockdown CAFs also reduced the percentage of GATA3 (Th2 transcription factor) positive T cells and increased the percentage of T-bet (Th1 transcription factor) positive T cells **(D-G)**. ELISA assay indicated that CTHRC1 knockdown CAFs promoted IL-4 production in culture medium **(H)**. **(I-K)**, CTHRC1 knockdown CAFs reduced the percentage of CD206^+^ M2 macrophage and expression of M2 macrophage markers (CD206, Ym1, ARG1, TGF-β), and increased the percentage of CD80^+^ M1 macrophage and expression M1 macrophage markers (CD80, NOS2, TNF-α, CXCL9) of mouse BMDMs. L-S, human naïve T cells were activated and co-cultured with CTHRC1 knockdown H-CAFs5 cells indirectly using a transwell system or directly in 24-well plates. Flow cytometric analysis indicated that CTHRC1 knockdown CAFs promoted Th1 (IFN-γ^+^) differentiation and suppressed Th2 (IL-4^+^) differentiation of human naïve T cells **(L-N)**. CTHRC1 knockdown CAFs also reduced the percentage of GATA3 (Th2 transcription factor) positive T cells and increased the percentage of T-bet (Th1 transcription factor) positive T cells **(O-R)**. ELISA assay indicated that CTHRC1 knockdown CAFs reduced IL-4 production in culture medium **(S)**. **(T-V)** CTHRC1 knockdown CAFs reduced the percentage of CD206^+^ M2 macrophage and expression of M2 macrophage markers (CD206, Ym1, ARG1, TGF-β), and increased the percentage of CD80^+^ M1 macrophage and expression M1 macrophage markers (CD80, NOS2, TNF-α, CXCL9) of human THP1 cells. **(W)** Western blot analysis of the PI3K/Akt and NF-κB signaling pathways in human (H-CAFs5) and mouse (M-CAFs1) CAFs following CTHRC1 knockdown. GAPDH served as the loading control. **(X)** Quantification of immunosuppressive cytokine secretion (TGF-β, IL-6, IL-10, CCL2, CXCL12) in the supernatants of H-CAFs5 and M-CAFs1 cells measured by ELISA. Data are presented as mean ± SD; *p < 0.05, **p < 0.01, ***p < 0.001 indicate statistical significance.

Next, we performed loss-of-function assays. Naïve T cells from C57BL/6 mice were activated and co-cultured with CTHRC1-depleted M-CAFs1 cells, either via a transwell system or in direct contact cultures. Conversely, CTHRC1 depletion in M-CAFs1 promoted Th1 differentiation while suppressing Th2 polarization in mouse T cells (**Figures 6A–H**). A similar shift was observed in BMDMs, wherein CTHRC1 knockdown facilitated M1 polarization while restraining M2 programming (**Figures 6I–K**). These findings were recapitulated in human cellular contexts: silencing CTHRC1 in H-CAFs5 promoted Th1 differentiation in human T cells (**Figures 6L–S**) and drove M1 polarization in THP-1 monocytes (**Figures 6T–V**). Mechanistically, CTHRC1 depletion markedly suppressed the phosphorylation of PI3K, Akt, and p65, confirming that CTHRC1 is a critical positive regulator of the PI3K/Akt/NF-κB signaling cascade (**Figure 6W**). Accordingly, CTHRC1 knockdown significantly attenuated the secretion of key cytokines, including TGF-β, IL-6, IL-10, CCL2, and CXCL12 (**Figure 6X**). Among these cytokines, IL-6 is a bona fide downstream target of PI3K/Akt/NF-κB signaling and plays important role in macrophage polarization and Th2 cell differentiation (27, 28). To evaluate the role of IL-6 in CTHRC1^+^ CAFs-mediated Th2 differentiation and M2 macrophage polarization, we depleted IL-6 in culture medium using an antibody against IL-6 (αIL6). Our data indicated that depletion of IL-6 almost abrogated the influence of CTHRC1 overexpression on Th2 differentiation and M2 macrophage polarization *in vitro* (**Supplementary Figure 5**). Collectively, these data establish that CTHRC1^+^ CAFs drive Th2 differentiation and M2 macrophage polarization *in vitro*.

### 3.5 CTHRC1^+^ CAFs suppress antitumor T cell activity in bladder cancer xenografts

**Figure 7:**
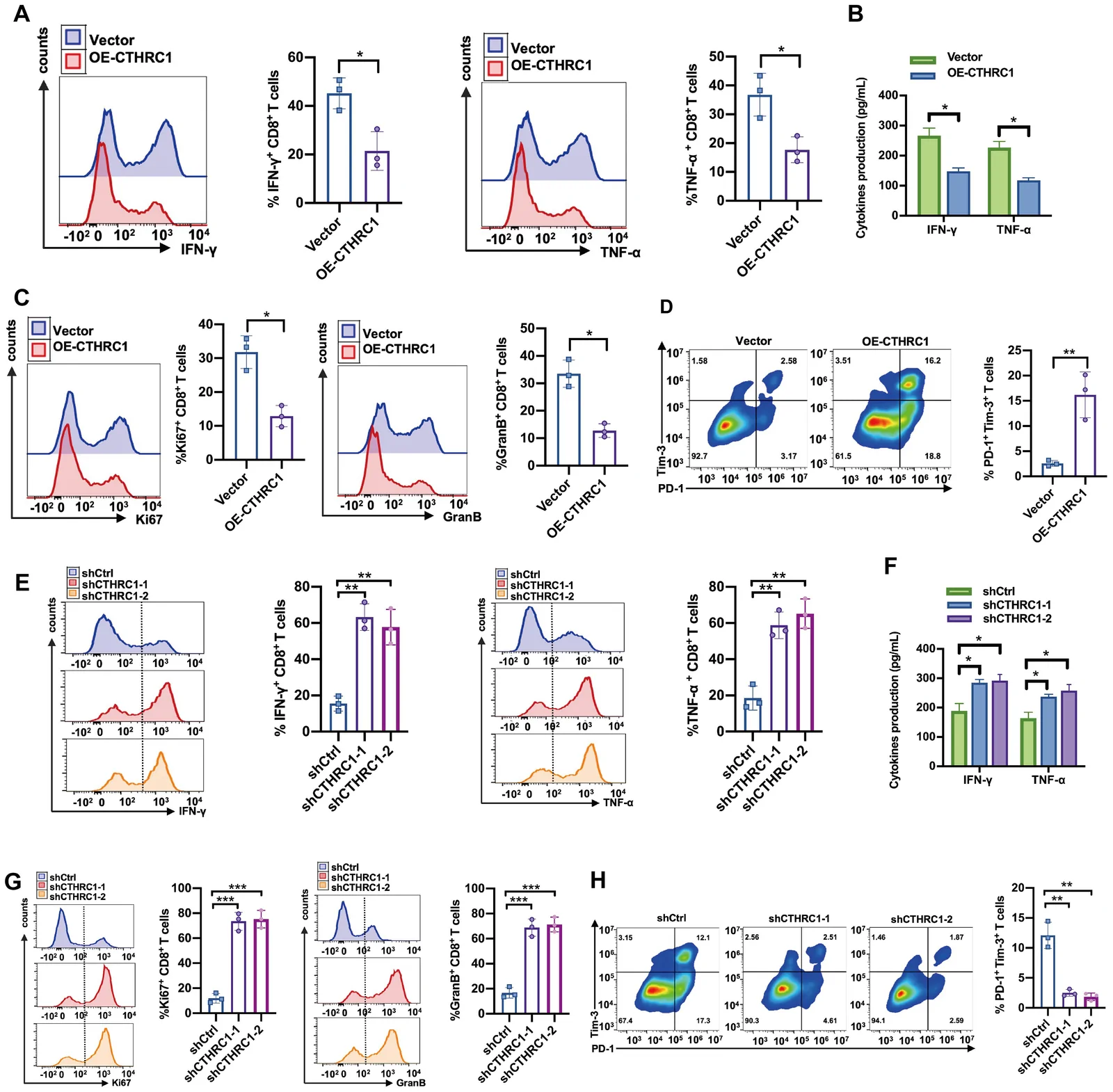
CTHRC1^+^ CAFs suppress antitumor CD8^+^ T cell activity in bladder cancer xenografts. **(A-D)**. MB49 and CTHRC1-overexpression M-CAFs3 cells (1: 1) were mixed together and subcutaneously implanted into C57BL/6 mice for three weeks. Flow cytometric analysis indicated that CTHRC1 overexpression CAFs suppressed the infiltrating of IFN-γ^+^ CD8^+^ and TNF-α^+^ CD8^+^ T cells in MB49 tumors **(A)**. ELISA assay indicated CTHRC1 overexpression CAFs reduced IFN-γ and TNF-α production in MB49 tumors **(B)**. Representative histograms indicated that CTHRC1 overexpression CAFs reduced the frequency of Ki67+ and Granzyme B+ infiltrating CD8^+^ T cells, and increased the percentages of infiltrating PD-1^+^Tim-3^+^ CD8^+^ T cells in MB49 tumors **(C, D)**. **(E-H)** MB49 and CTHRC1 knockdown M-CAFs1 cells (1: 1) were mixed together and subcutaneously implanted into C57BL/6 mice. Flow cytometric analysis indicated that CTHRC1 knockdown CAFs increased the infiltrating of IFN-γ^+^ CD8^+^ and TNF-α^+^ CD8^+^ T cells in MB49 tumors **(E)**. ELISA assay indicated CTHRC1 overexpression CAFs increased IFN-γ and TNF-α production in MB49 tumors **(F)**. Representative histograms indicated that CTHRC1 knockdown CAFs increased the frequency of Ki67^+^ and Granzyme B+ infiltrating CD8^+^ T cells, and decreased the percentages of infiltrating PD-1^+^Tim-3^+^ CD8^+^ T cells in MB49 tumors **(G, H)**. Data are presented as mean ± SD; *p < 0.05, **p < 0.01, ***p < 0.001 indicate statistical significance.

Accumulated studies have established macrophages and T helper (Th) cells as pivotal regulators of CD8^+^ T cell activity within the tumor microenvironment (29). Thus, we speculated that CTHRC1^+^ CAFs might affect antitumor T cell activity in bladder cancer. IFN-γ and TNF-α are key effectors mediating the cytotoxic functions of CD8^+^ T lymphocytes. Compared with vector-transduced controls, overexpression of CTHRC1 in M-CAFs3 significantly reduced the frequency of IFN-γ^+^ and TNF-α^+^ CD8^+^ T cells in MB49 tumors and diminished intratumoral IFN-γ and TNF-α production (**Figures 7A, B**). Ki67 and granzyme B denote proliferative capacity and effector functionality in CD8^+^ T cells, respectively. CTHRC1-overexpressing M-CAFs3 significantly reduced the frequency of both Ki67^+^ and granzyme B^+^ CD8^+^ T cells within MB49 tumors (**Figure 7C**). In the context of cancer progression, PD-1 and Tim-3 dual positivity serves as a hallmark of T cell exhaustion. Accordingly, CTHRC1 overexpression in M-CAFs3 markedly elevated the frequency of this exhausted CD8^+^ T cell subset in MB49 tumors (**Figure 7D**). Conversely, CTHRC1 depletion in M-CAFs1 cells yielded opposing effects: compared with shCtrl-transduced controls, CTHRC1 knockdown elevated the frequencies of IFN-γ^+^ and TNF-α^+^ CD8^+^ T cells and enhanced cytokine production (**Figures 7E, F**), while upregulating Ki67^+^ and granzyme B^+^ subsets and downregulating PD-1^+^Tim-3^+^ exhausted T cells (**Figures 7G, H**).

To assess the functional contribution of Th2 and M2 cells to CTHRC1^+^ CAF-mediated immunosuppression, we selectively depleted these subsets in MB49 tumors using anti-IL-4 (αIL4) and anti-CD206 (αCD206) antibodies, respectively (**Supplementary Figures 6A,B**). Depletion of Th2 cells or M2 macrophages led to a significant expansion of IFN-γ^+^, TNF-α^+^, Ki67^+^, and granzyme B^+^ CD8^+^ T cells, coupled with a marked reduction in the PD-1^+^Tim-3^+^ exhausted subset within MB49 tumors (**Supplementary Figures 6C–F**). To evaluate whether CAFs directly suppressed the antitumor activity of CD8^+^ T cells, we co-cultured CTHRC1-overexpressing M-CAFs3 with CD8^+^ T cells isolated from peripheral blood of C57BL/6 mice in a transwell system. Our results indicated that co-cultured with CTHRC1-overexpressing M-CAFs3 showed no influence on the frequency of IFN-γ^+^ and TNF-α^+^ CD8^+^ T cells (**Supplementary Figures 7A,B**), Ki67^+^ and granzyme B^+^ CD8^+^ T cells (**Supplementary Figure 7C**), or PD-1^+^Tim-3^+^ exhausted T cells (**Supplementary Figure 7D**) compared with vector transduced M-CAFs3 cells. Collectively, these findings demonstrate that CTHRC1^+^ CAFs suppress antitumor T cell immunity in bladder cancer xenografts.

## 4 Discussion

Metastasis, a leading cause of cancer-related mortality, is governed by a multitude of factors, including protein-coding genes and non-coding RNAs (30–32). Emerging evidence indicates that CTHRC1 is aberrantly expressed across diverse malignancies and plays a pivotal role in cancer progression. For instance, CTHRC1 serves as a prognostic biomarker in gastrointestinal stromal tumor (GIST) patients and potentiates cellular invasiveness via activation of the Wnt/PCP-Rho signaling pathway (33). In colorectal cancer, CTHRC1 predicts poor survival of cancer patients and promotes cell motility of colorectal cancer cells via induing epithelial-mesenchymal transition (34). In gastric cancer, CTHRC1 is transcriptionally upregulated through promoter demethylation and TGF-β1 stimulation, thereby facilitating cancer cell invasion (35). Notably, fibroblasts serve as the major cellular origin of CTHRC1, mediating crosstalk within the tumor microenvironment to orchestrate critical biological processes. For instance, CTHRC1 drives the activation of pancreatic stellate cells (PSCs) into myofibroblast-like CAFs and facilitates pancreatic tumor proliferation and metastasis through the transcriptional upregulation of POSTN (36). CTHRC1 contributes to keloid pathogenesis by attenuating collagen synthesis and fibroblast migration through suppression of the TGF-β/Smad pathway and inhibition of YAP nuclear translocation (37). In hepatocellular carcinoma (HCC), senescent fibroblasts secrete CTHRC1, which drives cancer stemness and metastasis through the SOX4/CTHRC1/Notch1 signaling axis (38). In lung cancer, CTHRC1^+^ CAFs are enriched in therapy-resistant tumors and drive glycolytic reprogramming of cancer cells via activation of the TGF-β/Smad3 signaling pathway (39). In this study, we isolated CAFs from bladder cancer tissues and demonstrated that the CTHRC1^+^ subset promotes bladder cancer cell proliferation, migration, and invasion both *in vitro* and *in vivo*. These data establish CTHRC1 as a critical driver of bladder cancer progression.

In this study, CTHRC1^+^ CAFs promoted the infiltration of immunosuppressive M2 macrophages and Th2 cells in bladder cancer xenografts *in vivo*, while facilitating Th2 cell differentiation and M2 macrophage polarization *in vitro*. M2 macrophages and Th2 cells serve as pivotal immunosuppressive regulators within the tumor microenvironment. Accumulating evidence implicates CTHRC1^+^ CAFs in orchestrating an immunosuppressive niche across diverse malignancies. For instance, CTHRC1 drives M2 macrophage polarization in endometrial carcinoma via activation of the integrin β3-Akt signaling pathway (40). Moreover, CTHRC1^+^ CAFs and SPP1^+^ TAMs synergize to foster a pro-tumorigenic niche by driving extracellular matrix deposition and epithelial-mesenchymal transition (EMT). Moreover, CTHRC1^+^ CAFs and SPP1^+^ TAMs synergize to foster a pro-tumorigenic niche by driving extracellular matrix deposition and epithelial-mesenchymal transition (EMT) (41). In colorectal cancer, CTHRC1 enhances macrophage chemotaxis through upregulation of CCL15 (42). In prostate cancer, the tumor microenvironment shows increasing immunosuppressive CTHRC1^+^ CAFs together with fewer T cells, increased T cell dysfunction, and higher TAM levels (43). Furthermore, M2 macrophages serve as key orchestrators of the Th2 immune landscape. They orchestrate the Th2 response while concurrently dampening Th1 immunity through a sophisticated interplay of cytokine secretion and metabolic rewiring, thereby sustaining an immunosuppressive tumor microenvironment. This may account for the concurrent promotion of M2 macrophage polarization and Th2 differentiation by CTHRC1^+^ CAFs in bladder cancer.

CTHRC1 exerts its pro-tumorigenic effects by directly activating or modulating core signal transduction networks within both cancer cells and stromal cells. Notably, the Wnt signaling cascade ranks among the most frequently dysregulated pathways in human cancers, with CTHRC1 emerging as a critical regulator of this network (14, 44, 45). The TGF-β pathway exhibits a dual role in carcinogenesis, CTHRC1 strongly biases this signaling toward a tumor-promoting phenotype (15, 46). Moreover, CTHRC1 potentiates cancer cell adhesion to the extracellular matrix through integrin interaction and subsequent activation of the Src/FAK pathways (47, 48). In the present study, we identified that CTHRC1 activates the PI3K/Akt/NF-κB signaling axis while regulating cytokine secretion, thereby facilitating Th2 cell differentiation and M2 macrophage polarization. Critically, these pathways do not operate in isolation but are functionally intertwined, as Wnt, TGF-β, and Src/FAK signaling exhibit significant interconnectivity with the PI3K/Akt/NF-κB cascade (49, 50). Complementing these findings, CTHRC1 has been implicated in PI3K/Akt activation across diverse malignancies. For instance, it drives the proliferation of Wilms’ tumor cells by stimulating this pathway (51). Similarly, CTHRC1 orchestrates tumor growth and metastasis through modulation of the PI3K/Akt axis in hepatocellular carcinoma (52).

In the present study, CTHRC1 overexpression in CAFs activated the PI3K/Akt/NF-κB pathway and drove robust secretion of TGF-β, IL-6, IL-10, CCL2 and CXCL12, whereas CTHRC1 knockdown produced the opposite effect. Prior work by Li et al. demonstrated that CTHRC1 promotes bladder cancer cell proliferation and invasion through the PI3K/Akt pathway when CTHRC1 is expressed intrinsically within the tumor cells (53). Our study extends this established axis into the stromal compartment. We showed that CTHRC1, when expressed in CAFs, activated PI3K/Akt signaling within the fibroblast and further engaged NF-κB downstream. This CAF-intrinsic activation drove robust secretion of TGF-β, IL-6, IL-10, CCL2 and CXCL12. These secreted factors are not only immunosuppressive mediators but also direct-acting paracrine effectors on bladder cancer cells. For example, accumulating evidence indicates that CAF-derived TGF-β1 promotes bladder cancer cell migration, invasion and EMT via the FAP/VCAN axis and PI3K/AKT signaling (54). CAF-secreted IL-6 induces EMT, proliferation, migration and invasion of bladder cancer cells through STAT3 and AKT pathway activation (55). CAF-derived CXCL12 directly enhances bladder cancer cell proliferation, invasion and migration via CXCR4 (56). Likewise, CCL2-CCR2 signaling facilitates tumor cell migration through PKC-dependent paxillin phosphorylation, and TGF-β1, IL-6 and CXCL12 collectively orchestrate EMT-related transcriptional reprogramming (57). Therefore, the same CTHRC1-driven secretome that polarizes macrophages toward an M2 phenotype and biases CD4^+^ T cells toward Th2 differentiation concurrently establishes a paracrine loop that directly stimulates bladder cancer cell growth and motility. A notable limitation of our study is that the selection of CTHRC1-high (H-CAFs5, M-CAFs1) and CTHRC1-low (H-CAFs1, M-CAFs3) CAF strains is based on Western blot screening (**Figure 1J**), which may introduce selection bias. Moreover, the spatial co-localization of CTHRC1^+^ CAFs with Macrophage/Th2/CD8^+^ T cells in bladder cancer tissues is not demonstrated in our study, which are warranted in our future study.

An important question raised by our study is whether CTHRC1 expression in CAFs arises from an intrinsic, fibroblast-autonomous program or is induced by extrinsic signals from the tumor microenvironment. Our data show that CTHRC1 is highly expressed in CAFs isolated from bladder cancer tissues and mouse xenografts but is barely detectable in paired normal fibroblasts, suggesting that the tumor microenvironment is necessary for CTHRC1 upregulation. Several lines of evidence support the notion that tumor-derived factors, particularly TGF-β1, are major inducers of CTHRC1 in fibroblasts. In gastric cancer, CTHRC1 is transcriptionally upregulated by TGF-β1 via Smad signaling (35). In pancreatic cancer, TGF-β1 secreted by cancer cells drives CTHRC1 expression in pancreatic stellate cells, promoting their transdifferentiation into myofibroblast-like CAFs (36). In hepatocellular carcinoma, senescent fibroblasts secrete CTHRC1 in response to tumor-derived cues (38). On the other hand, we cannot exclude a fibroblast-autonomous component. Once CTHRC1 is induced, it activates PI3K/Akt/NF-κB signaling in CAFs and stimulates autocrine secretion of TGF-β, IL-6, and other factors. This creates a positive feedback loop in which CTHRC1 itself perpetuates its own expression and reinforces the CAF phenotype, as reported in fibrotic diseases and cancer (11, 46). Thus, CTHRC1 likely operates through a two-phase model, which it is initial induced by tumor-derived TGF-β family ligands, and followed by self-sustaining autocrine/paracrine maintenance within the CAF population. However, more evidence is needed to prove this.

## 5 Conclusion

In conclusion, we found that CTHRC1 was predominantly expressed in CAFs of bladder cancer, while CTHRC1^+^ CAFs facilitated growth, migration and invasion of bladder cancer cells *in vitro* and *in vivo*. Moreover, CTHRC1^+^ CAFs induced the infiltration of immunosuppressive M2 macrophage and Th2 cells, thus suppressed antitumor T cell activity in bladder cancer xenografts. Our data elucidated a novel role of CTHRC1 in regulating M2 macrophage polarization and Th2 bias in bladder cancer.

## Data Availability

The original contributions presented in the study are included in the article/**Supplementary Material**. Further inquiries can be directed to the corresponding author.
