# Supplementary material for: CTHRC1+ cancer associated-fibroblasts promotes malignant progression of bladder cancer cells via inducing an immunosuppressive tumor microenvironment
Source: Front Immunol. 2026 Aug 12;17:1898002. doi: 10.3389/fimmu.2026.1898002 (PMC13506716; doi:10.3389/fimmu.2026.1898002)
Supplement: Supplementary file 1 [file DataSheet1.docx]

Supplementary Material

# 1 Supplementary Figures

Figure S1. The gating strategy in flow cytometry analysis.

Figure S2. Pan-cancer analysis of CTHRC1 expression using TIMER database.

Figure S3. Correlation of CTHRC1 expression with infiltrating immune cells in bladder cancer patients. A-H, Correlation of CTHRC1 expression with infiltrating macrophage (A), M1 macrophage (B), M2 macrophage (C), MDSC (D), Th1 (E), Th2 (F), Tregs (G) and CD8^+^ T (H) cells in bladder cancer patients.

Figure S4. Influence of PI3K and Akt inhibitors on CTHRC1-mediated Th2 and M2 polarization *in vitro*. A-E. Mouse naïve T cells were activated and co-cultured with CTHRC1-overexpressing M-CAFs3 cells indirectly using a Transwell system. Flow cytometric analysis showed that CTHRC1 overexpression CAFs reduced the percentage of T-bet⁺ Th1 cells (A, B) and increased the percentage of GATA3⁺ Th2 cells (C, D), while these effects were reversed by treatment with the PI3K inhibitor LY294002 or the Akt inhibitor MK-2206. ELISA analysis further confirmed that CTHRC1-overexpression CAFs enhanced IL-4 secretion in the culture medium, an effect that was significantly attenuated by both inhibitors (E). F-H. In mouse bone marrow-derived macrophages (BMDMs) co-cultured with OE-CTHRC1 MCAFs3, flow cytometry revealed decreased percentages of CD80⁺ M1 macrophages and increased percentages of CD206⁺ M2 macrophages (F, G), along with downregulated mRNA expression of M1 markers (CD80, NOS2, TNF-α, CXCL9) and upregulated mRNA expression of M2 markers (CD206, Ym1, ARG1, TGF-β) (H). These phenotypic and transcriptional changes were largely abrogated by LY294002 or MK-2206 treatment. I. Western blot analysis of PI3K/Akt/NF-κB signaling pathway components (p-PI3K, PI3K, p-Akt, Akt, p-p65, p65) in M-CAFs3 cells with or without CTHRC1 overexpression and inhibitor treatment. GAPDH served as the loading control. J. Quantification of immunosuppressive cytokine secretion (TGF-β, IL-6, IL-10, CCL2, CXCL12) in supernatants from M-CAFs3 cells measured by ELISA. Data are presented as mean ± SD; *p < 0.05, **p < 0.01, ***p < 0.001 indicate statistical significance.

Figure S5. CTHRC1^+^ CAFs facilitate Th2 cell differentiation and M2 macrophage polarization via IL-6. A-E. Mouse naïve T cells were activated and co-cultured with CTHRC1-overexpressing M-CAFs3 cells indirectly using a Transwell system. Flow cytometric analysis showed that CTHRC1 overexpression CAFs reduced the percentage of T-bet⁺ Th1 cells (A, B) and increased the percentage of GATA3⁺ Th2 cells (C, D), while these effects were reversed by treatment with IL-6 antibody (αIL6). ELISA analysis further confirmed that CTHRC1-overexpression CAFs enhanced IL-4 secretion in the culture medium, an effect that was significantly attenuated by IL-6 antibody (E). F-H. In mouse bone marrow-derived macrophages (BMDMs) co-cultured with OE-CTHRC1 MCAFs3, flow cytometry revealed decreased percentages of CD80⁺ M1 macrophages and increased percentages of CD206⁺ M2 macrophages (F, G), along with downregulated mRNA expression of M1 markers (CD80, NOS2, TNF-α, CXCL9) and upregulated mRNA expression of M2 markers (CD206, Ym1, ARG1, TGF-β) (H). However, these effects were reversed by treatment with IL-6 antibody (αIL6). Data are presented as mean ± SD; *p < 0.05, **p < 0.01, ***p < 0.001 indicate statistical significance.

Figure S6. Depletion of Th2 cells and M2 macrophages restores anti-tumor T cell activity in MB49 tumors. A and B, MB49 tumor-bearing mice were treated with IgG, anti-IL-4 neutralizing antibody (αIL4) or anti-CD206 neutralizing antibody (αCD206) once a week for three times. Flow cytometric analysis indicated that the anti-IL-4 neutralizing antibody and anti-CD206 neutralizing antibody reduced the infiltrating of Th2 cells and CD206^+^ M2 macrophages in MB49 tumors. C-F, anti-IL-4 neutralizing antibody and anti-CD206 neutralizing antibody increased the percentage of IFN-γ^+^ , TNF-α^+^, Ki67^+^ and Granzyme B^+^ CD8^+^ infiltrating T cells, and reduced the percentage of infiltrating PD-1^+^Tim-3^+^ CD8^+^ T cells in MB49 tumors. Data are presented as mean ± SD; *p < 0.05, **p < 0.01, ***p < 0.001 indicate statistical significance.

Figure S7. CTHRC1-overexpressing CAFs shows no direct influence on antitumor CD8^+^ T cell activity in vitro. A-D, CTHRC1-overexpression M-CAFs3 cells or vector-transduced M-CAFs3 cells were co-cultured with CD8^+^ T cells (1: 1) isolated from peripheral blood of C57BL/6 mice in a transwell system. Flow cytometric analysis indicated that CTHRC1 overexpression CAFs showed no influence on the percentage of IFN-γ^+^ CD8^+^ and TNF-α^+^ CD8^+^ T cells (A), Ki67⁺ and granzyme B⁺ CD8⁺ T cells (C), or PD-1⁺Tim-3⁺ exhausted T cells (D) compared with vector transduced M-CAFs3 cells. ELISA assay indicated that CTHRC1 overexpression CAFs showed no influence on IFN-γ and TNF-α production of CD8^+^ T cells (B). ns= not significance.

# 2 Supplementary Tables

Supplementary Table 1. Primer sequences for qRT-PCR.

| Gene | Forward primer | Reverse primer |
| --- | --- | --- |
| Human VIM | GCTGCTAACTACCAAGACA | CTGTAGGTGGCAATCTCAA |
| Mouse Vim | GCAGCCTCTATTCCTCATC | AGTCCACCGAGTCTTGAA |
| Human FAP | GTCATTGCCTTGGTGGAT | GCTGTAATCTGGTCTTCAAC |
| Mouse Fap | TTCATACACAGCGACATACT | AGGCGACCAGCATAGATA |
| Human ACTA2 | CTTGAGAAGAGTTACGAGTTG | GATGCTGTTGTAGGTGGTT |
| Mouse Acta2 | TCATTGCTTCCTCCTCCT | CGTGTGAGAGTCTTGTGTA |
| Human FSP-1 | CAACTTGGACAGCAACAG | CCAACCACATCAGAGGAG |
| Mouse Pdgfrb | TTGTTGCTGTCCGTGTTAT | AGGTGCTGGAGATGTTGA |
| Human GAPDH | TATGACAACAGCCTCAAGAT | AGTCCTTCCACGATACCA |
| Mouse Gapdh | TCTCCTGCGACTTCAACA | TGTAGCCGTATTCATTGTCA |
| Human CD80 | GGAATACAACCAAGCAAGAG | GTCAGGCAGCATATCACA |
| Mouse Cd80 | GTGCTGCTGATTCGTCTT | GCCAGTAGATTCGGTCTTC |
| Human NOS2 | CGAGTCAGAGTCACCATC | CTCAGCCTGTACTTATCCAT |
| Mouse Nos2 | TACTGCTGGTGGTGACAA | CTGAAGGTGTGGTTGAGTT |
| Human TNF | GCAACAAGACCACCACTT | CTCCAGATTCCAGATGTCAG |
| Mouse Tnf | ACCACCATCAAGGACTCA | AGGTCTGAAGGTAGGAAGG |
| Human CXCL9 | AGTTCAAGTCCTGCCTCT | GCTGACAATCTGGTTGGA |
| Mouse Cxcl9 | TGCTACTACACCTGATTGC | CGCTGAAGATGTCTGAAGA |
| Human CD206 | AATGGCTTCGTGGAGAAC | GACCTTGGCTTCGTGATT |
| Mouse CD206 | GGCAAGTATCCACAGCAT | GTTCCATCACTCCACTCAA |
| Human Ym1 | TTCACTCTGGCTTCTTCTG | CCGAGGATTCTATGGACTG |
| Mouse Ym1 | CGTGACTATGAAGCATTGAA | TTCTGAGGAGTAGAGACCAT |
| Human ARG1 | AGTCAAGAAGAACGGAAGAA | GTGGTTGTCAGTGGAGTG |
| Mouse Arg1 | AAGGTCTCTACATCACAGAAG | CGAAGCAAGCCAAGGTTA |
| Human TGFB1 | CGACTACTACGCCAAGGA | GCTCTGATGTGTTGAAGAAC |
| Mouse Tgfb1 | ATTCCTGGCGTTACCTTG | CTGTATTCCGTCTCCTTGG |

Supplementary Table 2. Marker genes collected and curated for major cell-type annotation

| Cell type | Marker genes |
| --- | --- |
| Cancer cells | EPCAM，KRT7，KRT8，KRT18，KRT19，CDH1，UPK1A，UPK2，UPK3A，GATA3, KRT20, KRT5, KRT14，FGFR3, ELF3, CDKN1A, TSC1, ERRB2, TP63, EGFR, CD49, CD274 |
| Epithelial cells | EPCAM，KRT8， KRT18，KRT19，KRT7，KRT5， KRT14，KRT1，KRT10，CDH1 |
| Fibroblasts | FAP，PDPN, MMP2, PDGFRA, THY1 , MMP11, PDGFRL, TGFB3, COL1A2, DCN, COL3A1, COL6A1 |
| T cells | BCL11B, CD2, CD247, CD27, CD28, CD3D, CD3E, CD3G, CD6, CD7, CD8A, CD8B, CD96, CXCR6, FLT3LG, FYN, GIMAP4, GPR171, GZMK, GZMM, ICOS, ITK, LCK, LIME1, PRKCH, PSTPIP1, SH2D1A, SIRPG, TNFRSF9, TRAC, TRAT1, TRBC1, TRBC2, UBASH3A, ZAP70, AURKA, BIRC5, BUB1, CCNA2, CCNB1, CDC20, CDK1, DKN3, DKN1, HMGB2, MCM2, MCM5, MCM6, MYBL2, NUSAP1, PCNA, PLK1 |
| Endothelial cells | PECAM1, VWF, ENG |
| Myeloid cells | AIF1, APOBEC3A, AQP9, ASGR1, ASGR2, BST1 C5, AR1, CD1D, CD33, CD68, CDA, CFP, CHST15, CLEC4A, CLEC7A, CREB5, CSF3R, FAM198B, FCN1, FES, FOSB, FPR1, FZD2, HCK, HK3, HNMT, HPSE, IGSF6 L, ILRA2, LILRA3, LILRB2, LST1, MEFV, MNDA, MS4A6A, NCF2, NFE2, NLRP3, NOD2, P2RY13, PADI4, RNASE2, RNASE6, S100A12, SLC15A3, TLR2, TLR7, TLR8, UPK3A, VNN1, VNN2, ACP5, ADAMDEC1, BHLHE41, CCDC102B, CCL18, CCL22, CCL7, CHI3L1, COL8A2, CSF1, CXCL3, CXCL5, CYP27A1, DCSTAMP, GPC4, MARCO, MMP9, PLA2G7, PPBP, QPCT, SLAMF8, SLC12A8, TNFSF14, TREM2, ACHE, APOL3, APOL6, ARRB1, CCL19, CCL5, CCL8, CCR7, CD38, CD40, CLIC2, CXCL10, CXCL11, CXCL13, CXCL9, CYP27B1, DHX58, EBI3, GGT5, HESX1, IDO1, IFI44L, IL2RA, KIAA0754 |
| B cells | ABCB4, ADAM28, BACH2, BANK1, BCL7A, BEND5, BLK, BRAF, CD180, CD19, CD1C, CD22, CD37, CD69, CD72, CD79A, CD79B, CR2, CXCR5, EAF2, FAIM3, FCER2, FCGR2B, FCRL2, FRK, GPR18, GUSBP11, HHEX, HLA-DOB, IGHD, IGHM, IGKC, IGLL3P, IL4R, IRF8, KIAA0226L, LINC00921, LTB, LY86 MEP1A, MICAL3, MS4A1, NIPSNAP3B, NMBR, P2RX5, P2RY14, PNOC, PSG2, PTPRCAP, RALGPS2, RASGRP2, SELL, SIK1, SLC12A1, SPIB, STAP1, TCL1A, UGT1A8, VPREB3, ZNF286A , AIM2, ALOX5, CCR6, CD27, CLCA3P, DENND5B, FAM65B, GNG7, IFNA10, IL7, MBL2, NPIPB15, SIT1, SP140, TMEM156, TNFRSF13B, TNFRSF17, TRAF4, ZBTB32 |
| Mast cells | ATP8B4, BMP2K, BPI, C3AR1, CD33, CEACAM8, CLC, CMA1, CPA3, CRISP3, CTSG, FAM124B, FAM174B, FCER1A, GFI1, HDC, HPGDS, IL18R1, LTC4S, MS4A2, MS4A3, MYB, NOX3, NTRK1, P2RX1, P2RY14, PAQR5, PRG2, RAB27B, RGS13, SLC12A8, ST8SIA1, STAP1, STXBP6, TPSAB1, CCL1, CCL20, CCL4, CSF2 , CXCL3, GZMB, HOXA1, IL1A, IL1B, IL1RL1, IL3, IL5, LINC00597, TEC |
| NK cells | BPI, CAMP, CCL5, CD160, CD2, CD244, CD247, CD7, CD96, CDHR1, CEA, CAM8, CST7, CTSW, DEFA4, ELANE, GFI1, GNLY, GZMA , GZMB, GZMH, GZMK, GZMM, IL12RB2, IL18R1, IL18RAP, IL2RB, KIR2DL1, KIR3DL2, KLRB1, KLRC3, KLRC4, KLRD1, KLRF1, KLRK1, LCK, MGAM, MS4A3, NAALADL1, NKG7, NME8, PLEKHF1, PRF1, PRR5L, PTGDR, PTPRCAP, PVRIG, S1PR5, SH2D1A, TBX21, TEP1, TRBC1, TRDC, TTC38, TXK, ZAP70, ZNF135, APOBEC3G, APOL6, CCL4, CCND2, CD69, CDK6, CSF2, DPP4, FASLG, GPR171, GPR18, GRAP2, IFNG, KIR2DL4, KIR2DS4, LTA, LTB, NCR3, OSM, PTGER2, SOCS1, TNFSF14 |
| Neurons | SNAP25， SYT1，TUBB3，MAP2，STMN2， NEFL ，NEFM，NEFH，RBFOX3，SLC12A5 |
